# Irreversible Antagonists for the Adenosine A_2B_ Receptor †

**DOI:** 10.3390/molecules27123792

**Published:** 2022-06-13

**Authors:** Ahmed Temirak, Jonathan G. Schlegel, Jan H. Voss, Victoria J. Vaaßen, Christin Vielmuth, Tobias Claff, Christa E. Müller

**Affiliations:** PharmaCenter Bonn & Pharmaceutical Institute, Pharmaceutical & Medicinal Chemistry, University of Bonn, An der Immenburg 4, 53121 Bonn, Germany; ahmed.temirak@uni-bonn.de (A.T.); j.schlegel@uni-bonn.de (J.G.S.); jvoss@uni-bonn.de (J.H.V.); victoria.vaassen@uni-bonn.de (V.J.V.); cvielmut@uni-bonn.de (C.V.); tobias.claff@uni-bonn.de (T.C.)

**Keywords:** adenosine, BRET assay, covalent binding, Gα_15_, G protein activation, G protein-coupled receptor, mutagenesis, radioligand binding studies, synthesis, xanthine

## Abstract

Blockade of the adenosine A_2B_ receptor (A_2B_AR) represents a potential novel strategy for the immunotherapy of cancer. In the present study, we designed, synthesized, and characterized irreversible A_2B_AR antagonists based on an 8-*p*-sulfophenylxanthine scaffold. Irreversible binding was confirmed in radioligand binding and bioluminescence resonance energy transfer(BRET)-based Gα_15_ protein activation assays by performing ligand wash-out and kinetic experiments. *p*-(1-Propylxanthin-8-yl)benzene sulfonyl fluoride (**6a**, PSB-21500) was the most potent and selective irreversible A_2B_AR antagonist of the present series with an apparent K_i_ value of 10.6 nM at the human A_2B_AR and >38-fold selectivity versus the other AR subtypes. The corresponding 3-cyclopropyl-substituted xanthine derivative **6c** (PSB-21502) was similarly potent, but was non-selective versus A_1_- and A_2A_ARs. Attachment of a reactive sulfonyl fluoride group to an elongated xanthine 8-substituent (**12**, K_i_ 7.37 nM) resulted in a potent, selective, reversibly binding antagonist. Based on previous docking studies, the lysine residue K269^7.32^ was proposed to react with the covalent antagonists. However, the mutant K269L behaved similarly to the wildtype A_2B_AR, indicating that **6a** and related irreversible A_2B_AR antagonists do not interact with K269^7.32^. The new irreversible A_2B_AR antagonists will be useful tools and have the potential to be further developed as therapeutic drugs.

## 1. Introduction

**Figure 1:**
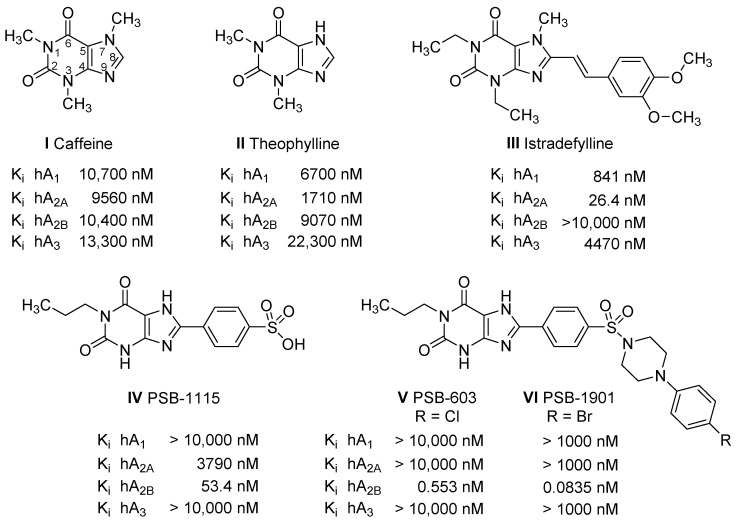
Chemical structures of selected AR antagonists (h = human) [18,23].

The nucleoside adenosine acts as an important extracellular signaling molecule in the body [1]. It activates G protein-coupled adenosine receptor (AR) subtypes (A_1_, A_2A_, A_2B_, and A_3_) and thereby transduces information across cell membranes. ARs have been recognized as promising drug targets. The non-selective AR antagonists caffeine (**I**) and theophylline (**II**), bioactive constituents of coffee and tea, have been used for thousands of years as central stimulants (see Figure 1) [2]. Caffeine is applied in combination with acetylsalicylic acid and/or paracetamol for the treatment of pain [3], while theophylline is utilized for the therapy of asthma and bronchial inflammation [4]. The anti-asthmatic, as well as the analgesic properties of caffeine and theophylline, are attributed, at least in part, to their A_2B_AR-blocking properties [5,6,7]. However, A_2B_-selective AR antagonists are not yet available as therapeutic drugs. The selective A_2A_AR antagonist istradefylline (**III**), a xanthine derivative derived from caffeine, is approved for the treatment of Parkinson’s disease in Asia and America (see Figure 1) [8]. Recently, the crucial role of adenosine in the innate immune system, being one of the most potent physiological immunosuppressive agents, has been recognized [9]. This effect is mediated by A_2A_- and A_2B_ARs expressed on immune cells. Moreover, A_2A_- and especially A_2B_ARs are often upregulated on cancer cells contributing to enhanced proliferation, metastasis, and angiogenesis [10,11,12]. Therefore, A_2A_- and A_2B_AR antagonists, including dual-acting compounds that block both AR subtypes, have been developed and are currently evaluated in clinical trials for cancer immunotherapy [13]. According to the Protein Data Bank (PDB) [14], 60 X-ray crystal structures of the human A_2A_AR in complex with 20 different antagonists [15] (and 5 agonists) have been published up to now, while structural data for the A_2B_AR are still elusive. However, X-ray co-crystal structures of the A_2B_AR in complex with antagonists would be highly useful in order to understand their binding and interactions. This knowledge would significantly support drug development efforts. Ligands that bind irreversibly to a receptor showing “infinite residence time” typically display particularly high stabilization of the purified protein [16,17].

Our previous work focused on the design, synthesis, and structure-activity relationships of A_2B_-selective AR antagonists as pharmacological tool compounds [18]. A milestone was the development of 1-propyl-8-(*p*-sulfophenyl)xanthine (PSB-1115, **IV**), the first water-soluble A_2B_AR antagonist showing good potency, but moderate selectivity, especially in rodents [19,20]. Nevertheless, it has been useful for studying the role of the A_2B_AR in vivo [21]. Based on the structure of PSB-1115, we obtained sulfonamide derivatives, e.g., PSB-603 and PSB-1901 (**V** and **VI**, see Figure 1), which displayed high potency combined with outstanding A_2B_-selectivity [18,22].

In the present study, we set out to design and prepare irreversible A_2B_AR antagonists based on PSB-1115, PSB-603, and related 8-(*p*-sulfophenyl)xanthine derivatives. Such compounds are required and will be useful for different purposes: (i) for stabilization of the isolated and purified A_2B_AR protein to allow crystallization and X-ray structure determination, and (ii) for potential therapeutic application especially in cancer (immuno)therapy resulting in long-lasting receptor blockade.

## 2. Results

### 2.1. Compound Design

We previously established an A_2B_AR homology model and have already performed docking studies with several xanthine derivatives [18,24]. The docking pose of the related xanthine derivative PSB-1901 (**VI**, Figure 1), for example, suggested that K269^7.32^ may function as a hydrogen bonding interaction partner of the sulfonamide [18] (amino acid superscript numbers represent the helix position according to the Ballesteros-Weinstein-System [25]). Hence, we hypothesized that the flexible nucleophilic side chain of K269^7.32^ may also represent a possible covalent reaction partner, e.g., for a sulfonyl fluoride function. Sulfonyl fluoride moieties have successfully been utilized for preparing irreversible ligands for A_1_—[17], A_2A_—[26], and A_3_ARs [27] targeting lysine or tyrosine residues. Irreversible ligands for the A_2B_AR have not yet been described.

**Figure 2:**
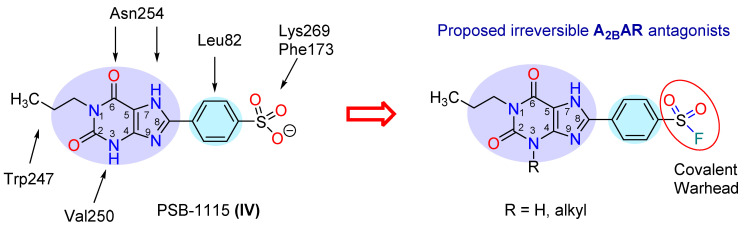
Design of irreversible A_2B_AR antagonists based on a published docking pose [18].

In this project, we optimized the structure of PSB-1115 (**IV**, Figure 2), one of the early selective A_2B_AR antagonists having a terminal sulfonate residue [20,28]. We kept the main pharmacophoric features essential for important interactions with the A_2B_AR, namely the flat xanthine core having a propyl substitution at the *N*1-position, a hydrogen bond acceptor group (*C*6-carbonyl), a hydrogen bond donating group (*N*7-hydrogen) and a phenyl ring attached to the 8-position of xanthine. The main modification is the replacement of the sulfonic acid moiety by the potentially covalent warhead sulfonyl fluoride, which was reported to irreversibly bind to several amino acids including lysine residues. Also, alkyl residues were introduced at the *N*3-position of the xanthine core to improve the physicochemical properties, in particular the aqueous solubility of the targeted compounds. Moreover, we considered an extended scaffold, similar to PSB-603 and PSB-1901 (**V**, **VI**), to evaluate different lengths of the linker between the xanthine core and the reactive warhead, since a longer linker might be suitable for interaction with residues located distant from the orthosteric binding pocket, close to the extracellular domains. Additionally, antagonists bearing longer lipophilic substituents at position 8 of the xanthine core, such as PSB-603, PSB-1901, and MRS-1754, displayed higher affinity as well as selectivity towards A_2B_ARs versus other AR subtypes.

### 2.2. Syntheses

**Scheme 1:**
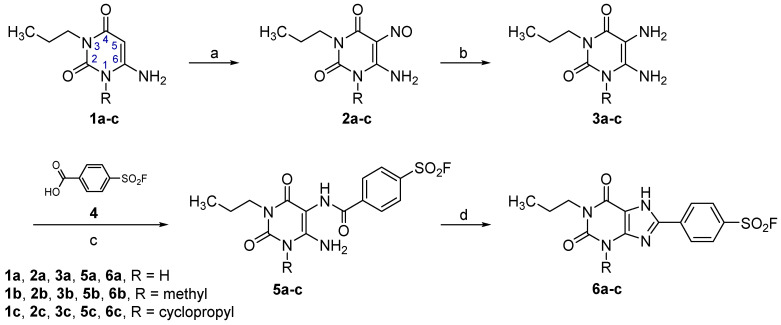
Preparation of compounds **6a–c** ^1^. ^1^ Reagents and conditions: (a) NaNO_2_, AcOH (50% aq), 65 °C, 10 min, 81–94%; (b) Na_2_S_2_O_4_, NH_3_ (12.5% aq), 65 °C, 10 min, 68–80%; (c) EDC, DMF, room temperature (rt), 2–4 h, 66–73%; (d) PPSE, (1) 120 °C, 10 min; (2) 170 °C, 2 h, 52–68%.

**Scheme 2:**
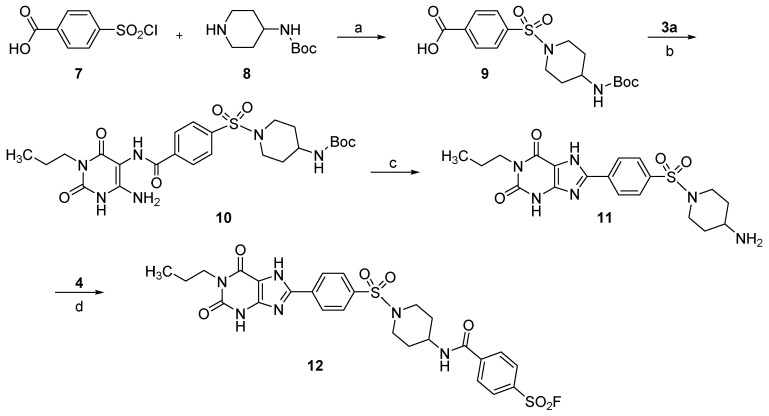
Preparation of compound **12** ^1^. ^1^ Reagents and conditions: (a) TEA, dioxane, rt, 36 h, 70%; (b) EDC, DMF, rt, 3 h, 68%; (c) PPSE, (1) 120 °C, 10 min; (2) 170 °C, 4 h, 60%; (d) HATU, DIPEA, DMF, rt, 18 h, 30%.

The synthetic routes to obtain the target compounds are depicted in Scheme 1 and Scheme 2. Nitrosation and subsequent reduction of the previously reported 3-propyl-6-aminouracil derivatives (**1a**–**c**) [29,30,31] was performed as described [30] yielding the diaminouracils **3a**–**c**. Amide coupling of **3a**–**c** with the 4-(fluorosulfonyl)benzoic acid (**4**) using the coupling reagent *N*-ethyl-*N′*-(3-dimethylaminopropyl)carbodiimide hydrochloride (EDC) [19,22] afforded the uracil carboxamide derivatives **5a**–**c**. Final ring closure reaction with polyphosphoric acid trimethylsilyl ester (PPSE) [20,28] afforded the target compounds **6a**–**c**.

As a second approach, we prepared a xanthine-derived antagonist (target compound **12**, Scheme 2) with an extended substituent in the 8-position and a terminal sulfonyl fluoride warhead, that would be closer to the extracellular loops (ECLs) of the A_2B_AR and might interact covalently with tyrosine or lysine residues [32]. The benzoic acid derivative **9** was prepared by reaction of 4-(chlorosulfonyl)benzoic acid (**7**) with *tert*-butylpiperidin-4-ylcarbamate (**8**) in the presence of triethylamine (TEA) as a base [33]. Subsequent amide coupling of **9** with the diaminouracil derivative **3a** afforded uracilcarboxamide derivative **10**. Interestingly, ring closure reaction using PPSE, according to our previous publication [28], resulted in a xanthine ring formation, and at the same time in deprotection of the *tert*-butyloxycarbonyl (Boc) protecting group yielding compound **11** with a free amino group. Amide coupling of **11** with 4-(fluorosulfonyl)benzoic acid (**4**) using the coupling reagent 1-[bis(dimethylamino)methylene]-1*H*-1,2,3-triazolo [4,5-b]pyridinium 3-oxo-hexafluorophosphate (HATU) afforded the final product **12**.

### 2.3. Pharmacological Characterization

#### 2.3.1. Radioligand Binding

The potentially irreversible xanthine derivatives were investigated for their binding affinity at human A_2B_ARs and for their selectivity versus the other human AR subtypes, A_1_, A_2A_, and A_3_, by competitive radioligand binding experiments. The reversible standard A_2B_AR antagonist PSB-1115 (**IV**), bearing a sulfonate group, was evaluated for comparison [19,28]. All new xanthine derivatives showed a low nanomolar apparent affinity for the A_2B_AR with high selectivity versus the A_3_AR subtype. Xanthine derivative **12**, bearing an extended 8-substituent, was the most potent A_2B_AR antagonist of the present series (apparent K_i_ value 7.37 nM); it also showed the highest selectivity of more than two orders of magnitude. This is well in agreement with analogous potent A_2B_AR antagonists [34], showing that certain large substituents in the xanthine 8-position can confer high A_2B_-selectivity presumably by engaging domains outside of the orthosteric site, e.g., in the extracellular loop 2 (ECL2). This region varies largely between receptor subtypes—in contrast to the orthosteric adenosine-binding site that shows a much higher degree of conservation [35,36].

The much smaller compound **6a** was found to be similarly potent (apparent K_i_ value 10.6 nM), and significantly more potent than the corresponding sulfophenylxanthine derivative PSB-1115 (14-fold difference). This might already be an indication of its irreversible binding. Moreover, it showed high selectivity, 38-fold against the A_1_AR, and 94-fold or higher against the A_2A_- and A_3_ARs.

Both, **6a** and **12**, are unsubstituted in the 3-position of the xanthine core, which had previously been shown to increase potency and selectivity [18,19,28], but may also reduce the water solubility of the compounds. As expected, the introduction of methyl or cyclopropyl into the xanthine 3-position slightly reduced potency (apparent K_i_ values: H, 10.6 nM; methyl, 15.9 nM; cyclopropyl, 26.9 nM) and diminished selectivity. The 3-methylxanthine derivative **6b** still displayed >18-fold selectivity, while the 3-cyclopropylxanthine derivative **6c** was virtually not selective anymore versus the A_1_- and A_2A_ARs (only 2- to 3-fold selectivity, see Table 1).

#### 2.3.2. Assessment of Reversible Versus Covalent Binding

The binding reversibility of the newly synthesized sulfonyl fluoride derivatives was investigated using ligand wash-out experiments. For this purpose, A_2B_AR-expressing membrane preparations of mammalian cells were incubated for 2 h at rt with the respective xanthine derivative. The cell membranes were subsequently washed extensively with buffer solution. Then, radioligand binding experiments were performed with the A_2B_-selective antagonist radioligand [^3^H]PSB-603 [22] in order to determine the (ir)reversibility of ligand binding (see Section 4).

**Figure 3:**
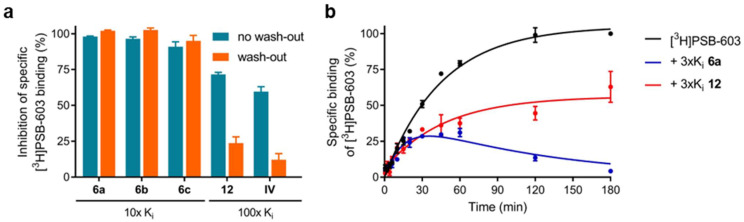
Assessment of reversibility of ligand binding. (**a**) Wash-out experiments after incubation of wildtype (wt) human A_2B_AR with compounds **6a–c**, **12** and **IV** at indicated concentrations. After an extensive washing procedure, specific binding of [^3^H]PSB-603 was determined. Data represents normalized mean values ± SEM from three independent experiments. (**b**) Competition association of [^3^H]PSB-603 alone (control) and in the presence of **6a** or **12** in concentrations representing 3-fold of the respective apparent K_i_ values. Values represent means ± SEM from two independent experiments.

As illustrated in Figure 3, the reversible A_2B_AR antagonist PSB-1115 (**IV**) inhibited the specific binding of [^3^H]PSB-603 prior to the wash-out procedure, but specific [^3^H]PSB-603 binding was no longer inhibited after wash-out of **IV**, which indicates reversible binding that can be washed out. Initial experiments with the most selective compound PSB-21503 (**12**) at an initial concentration corresponding to 10-fold of its K_i_ value resulted in an inhibition of [^3^H]PSB-603 binding of below 50% (data not shown). Please note that the given concentration refers to the concentration used for the incubation of the receptor preparation with the antagonist. In the subsequent radioligand binding studies, the antagonist concentration is 10-fold lower due to a 10-fold dilution (see Section 4). The observed result already hinted at reversible binding. Therefore, the concentration was increased to 100-fold of the K_i_ of **12**, in analogy to the conditions employed for investigating the reversible reference compound **IV**. Similar results were observed with the higher concentration indicating that PSB-21503 (**12**) binds reversibly to the A_2B_AR, like PSB-1115 (**IV**). In contrast, PSB-21500 (**6a**), PSB-21501 (**6b**), and PSB-21502 (**6c**), used in a 10-fold lower concentration (10-fold of their K_i_ value) still showed full inhibition of [^3^H]PSB-603 binding after an extensive membrane washing procedure. This indicates an irreversible binding mechanism for **6a**–**6c**.

Next, competition association experiments were performed highlighting the different behavior of the irreversible antagonist **6a** as compared to the reversible antagonist **12** (Figure 3b). The irreversible antagonist **6a** almost completely displaced [^3^H]PSB-603 with time, whereas **12** reached a binding equilibrium upon incubation with the radioligand. Thus, **6a** is characterized by an extremely slow or lacking dissociation providing further evidence for its irreversible binding mode.

#### 2.3.3. Functional Assays

**Figure 4:**
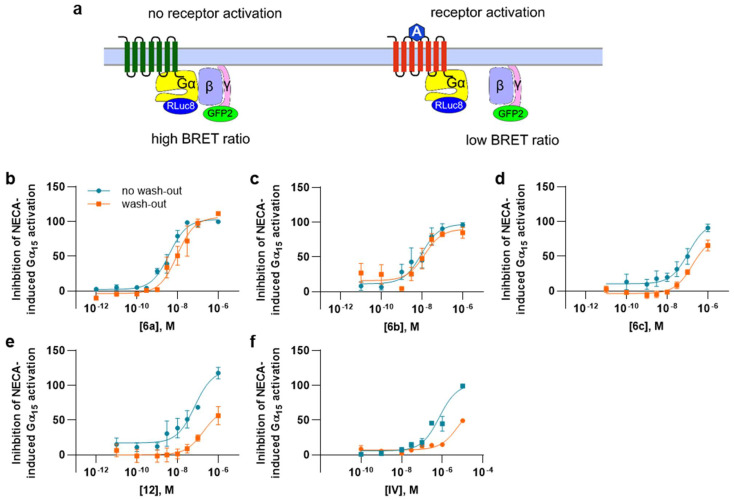
Functional assessment of A_2B_AR inhibition by the new antagonists**.** (**a**) Assay principle of the TRUPATH BRET² assay. When the receptor is not activated, the G protein (Gα_15_β_3_γ_13_) is in its inactive trimeric form, where Gα_15_-RLuc8 and Gγ_13-_GFP2 are in close proximity, resulting in a high BRET ratio. When the receptor is activated by an agonist (NECA). the trimer re-arranges, and the BRET ratio decreases due to a larger distance between the probes. (**b**–**f**) Concentration-response curves of (**b**) PSB-21500 (**6a**), (**c**) PSB-21501 (**6b**), (**d**) PSB-21502 (**6c**), (**e**) PSB-21503 (**12**), and (**f**) PSB-1115 (**IV**) showing the percent inhibition of NECA-induced Gα_15_ activation without or after wash-out. Wash-out did not result in significant differences for **6a**, **6b**, and **6c**. In contrast, significantly reduced inhibition of receptor activation was observed after wash-out of **12** and **IV**.

The inhibitory potency of the compounds on A_2B_AR activation was determined in G protein activation assays using *N*-ethylcarboxamidoadenosine (NECA), a stable analog of adenosine, as an agonist. In the assay, a bioluminescence resonance energy transfer (BRET) ratio between a renilla luciferase 8 (RLuc8)-tagged Gα_15_ protein—a key effector protein of the A_2B_AR [38]—and a green fluorescent protein 2 (GFP2)-tagged Gγ_13_ protein is measured [39] (Figure 4a). Upon receptor activation by NECA, the BRET ratio decreases. If the receptor has previously been treated with an A_2B_AR antagonist, activation by NECA is inhibited, and thus the G protein heterotrimer does not dissociate.

PSB-21500 (**6a**) fully inhibited NECA-induced Gα_15_ protein activation with an IC_50_ of 4.57 ± 1.26 nM (see Figure 4b). Compound **6a** still fully blocked the receptor after extensive washing with assay buffer, confirming irreversible binding. PSB-21503 (**12**) blocked the receptor with somewhat lower potency (IC_50_ = 59.2 ± 20.1 nM) in this functional assay; wash-out reduced the inhibition by roughly 50%, confirming reversible binding of **12** (Figure 4c). However, the compound does not appear to rapidly dissociate from the receptor. PSB-21501 (**6b**) and PSB-21502 (**6c**) inhibited the receptor with IC_50_ values of 15.3 ± 7.1 nM and 284 ± 220 nM, respectively (Figure 4d,e). Both ligands blocked the A_2B_AR activation irreversibly in functional assays. The reversible antagonist PSB-1115 displayed an IC_50_ value of 865 ± 415 nM and could be washed out completely (Figure 4f).

### 2.4. Extracellular Lysine K269^7.32^ as Potential Interaction Partner for Irreversible Binding of 6a (PSB-21500)

#### 2.4.1. Docking Studies of Compound **6a**

In order to probe the hypothesis that K269^7.32^ represents the irreversible anchor for the new *p*-sulfonyl fluorides, covalent docking was performed with **6a** and K269^7.32^ as potential reaction partner for nucleophilic substitution (see Section 4).

**Figure 5:**
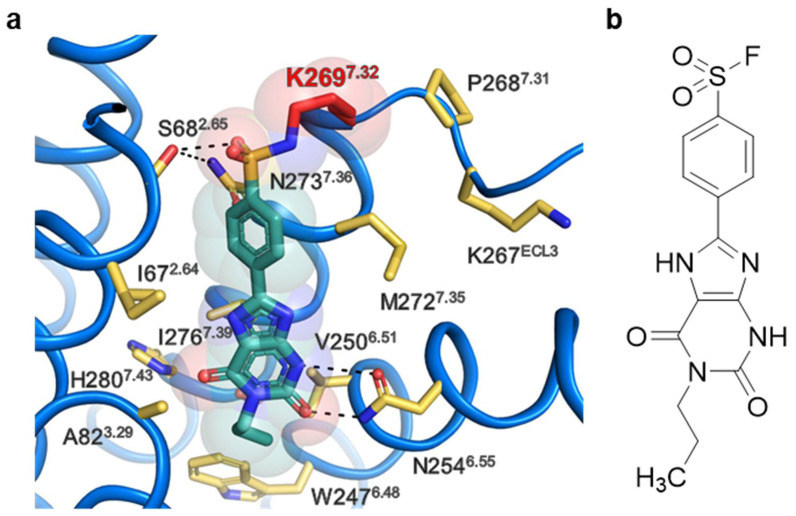
Suggested irreversible binding mode of PSB-21500 (**6a**) to the A_2B_AR. (**a**) Docking pose of **6a** in the previously published A_2B_AR homology model [18]. The lysine residue in position 269^7.32^ was chosen as an anchor for nucleophilic substitution. Covalent docking was performed using Maestro (Schroedinger). (**b**) Chemical structure of PSB-21500.

The binding pose of **6a** was observed inside the orthosteric binding pocket of the A_2B_AR. Direct hydrogen bonds of the C2-carbonyl and the *N*3 of **6a** to N254^6.55^ were observed (Figure 5). The xanthine scaffold of **6a** may be further stabilized by several hydrophobic contacts to I67^2.64^, V250^6.51^, and I276^7.39^ as well as by π-π stacking interactions to F173^ECL2^. The 8-phenyl moiety is rotated by approximately 40° and shows contacts to I67^2.64^ and M272^7.35^ according to the docked pose (Figure 5). The *p*-sulfonyl fluoride function is suggested to react with the amino group of K269^7.32^, but may also show hydrogen bonding to N273^7.36^ via S68^2.65^ (Figure 5).

#### 2.4.2. A_2B_AR Mutant K269^7.32^L

Docking experiments provided the plausible hypothesis that K269^7.32^ may serve as the covalent anchor for the irreversible A_2B_AR antagonist PSB-21500 (**6a**). To validate this assumption, mutagenesis of K269^7.32^ was performed. For this purpose, the lysine in position 269^7.32^ of the wt A_2B_AR was mutated to leucine (K269^7.32^L) which represents the respective residue in the A_2A_AR, the most closely related AR subtype. The new mutant was then subjected to additional pharmacological characterization using radioligand binding and functional experiments.

**Figure 6:**
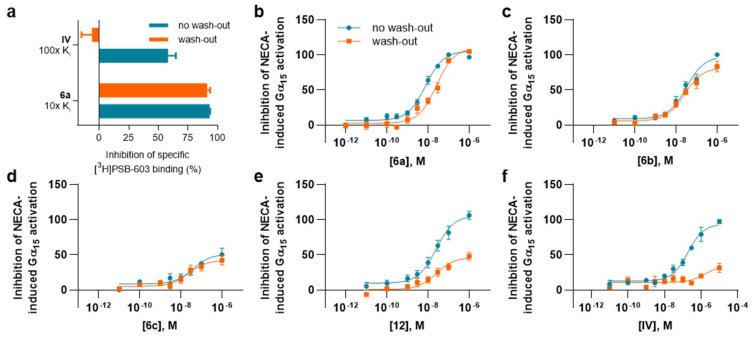
Binding and functional characterization of the A_2B_AR K269^7.32^L mutant. (**a**) Wash-out experiments after incubation of the A_2B_AR mutant with compounds **6a** and **IV** at 10-fold of the K_i_ value and at 100-fold of the K_i_ value, respectively. After an extensive washing procedure, the specific binding of [^3^H]PSB-603 was determined. Data represents normalized mean values ± SEM from three independent experiments. (**b**–**f**) Concentration-response curves for (**b**) PSB-21500 (**6a**), (**c**) PSB-21501 (**6b**), (**d**) PSB-21502 (**6c**), (**e**) PSB-21503 (**12**), and (**f**) PSB-1115 (**IV**) showing the percent inhibition of NECA-induced Gα_15_ activation by the investigated antagonists at the A_2B_AR K267^7.32^L mutant.

Although K269^7.32^ is presumed to be located inside the orthosteric ligand-binding pocket, the binding affinity of the A_2B_AR-selective radioligand [^3^H]PSB-603 [22] was virtually not affected (K_D_ 0.292 ± 0.034 vs. 0.553 ± 0.103 nM [22], *p* = 0.0739 using the unpaired *t*-test). Subsequently, wash-out experiments with the competitive A_2B_AR antagonist PSB-1115 (**IV**) and the irreversible antagonist PSB-21500 (**6a**) were carried out on the A_2B_AR mutant K269^7.32^L (Figure 6a). Much to our surprise, the wash-out experiments showed similar results as the analogous experiments at the wt A_2B_AR. PSB-1115 inhibited the specific binding of [^3^H]PSB-603, but wash-out abolished the effect. In contrast, the irreversible antagonist **6a** could not be washed-out at the mutant receptor showing the same behavior as it had displayed at the wt A_2B_AR. This indicates that PSB-21500 (**6a**) still binds irreversibly to the K269^7.32^L A_2B_AR mutant.

The same observation was made in functional assays performed on the K269^7.32^L mutant A_2B_AR. Each of the inhibitors displayed a similar inhibitory potency for the inhibition of NECA-induced Gα_15_ activation at the K269^7.32^L mutant compared to the wt A_2B_AR (Figure 6b–f). Moreover, PSB-21500 (**6a**), PSB-21501 (**6b**), and PSB-21502 (**6c**) could not be washed out, confirming the irreversible binding of these three compounds to the A_2B_AR. Again, washing decreased the A_2B_AR inhibition of PSB-21503 (**12**) by approximately 50%, and almost fully abrogated A_2B_AR inhibition by PSB-1115 (**IV**). It is worth mentioning that maximal inhibition of the NECA-activated K269^7.32^L A_2B_AR mutant by PSB-21502 (**6c**) was only 50%, both before and after wash-out (Figure 6d). In control experiments, it was, however, clearly shown that **6c** does not (partially) activate the mutant K269^7.32^L A_2B_AR (data not shown).

## 3. Discussion

Based on our previous work, we designed and developed the first irreversible A_2B_AR antagonists. The most potent and selective irreversible A_2B_AR antagonist is PSB-21500 (**6a**) displaying an apparent K_i_ value of 10.6 nM at the human A_2B_AR, 38-fold selectivity versus the A_1_AR, and about 100-fold selectivity or more versus the A_2A_- and A_3_AR subtypes. Irreversible binding was confirmed by wash-out experiments in radioligand binding studies as well as functional G protein activation assays. Docking studies suggested the lysine residue K269^7.32^ as the nucleophilic partner forming a stable covalent sulfonamide bond with the irreversibly binding xanthine derivatives. However, the K269^7.32^L mutation did not affect the potency of any of the inhibitors as determined in G protein activation assays. The binding of the A_2B_AR antagonists **6a**, **6b**, and **6c** remained irreversible in the mutant.

Interestingly, the 3-cyclopropyl-substituted xanthine derivative **6c** blocked only 50% of the NECA-induced Gα_15_ activation, which suggests an extremely slow reaction kinetic of **6c** at the K269^7.32^L A_2B_AR mutant. An alternative possible explanation, namely the partial-agonistic activity of **6c**, was excluded by control experiments.

Hence, we conclude that another residue is responsible for the anchoring of the ligand. K269^7.32^ is located at the extracellular ends of helix VII, close to the ECL3 of the A_2B_AR. The ECL3 itself contains two additional lysine residues (K265 and K267) that present potential reaction partners of the *p*-sulfonyl fluoride group (also refer to Figure 5). Our prediction of K269^7.32^ was based on an A_2B_AR homology model based on A_2A_AR crystal structures. However, ECLs are highly flexible and their correct structure is difficult to predict [40,41]. Thus, the ECL3 conformation in our A_2B_AR homology model may not be entirely correct and the above-mentioned alternative lysine residues may in fact be closer to the ligand-binding pocket and thus accessible to serving as a covalent anchor. Additionally, the K269^7.32^ was previously predicted to serve as a hydrogen bond interaction partner for the sulfonamide function of A_2B_AR antagonists [18] but the K269^7.32^L mutation did not alter the binding affinity of PSB-603 indicating a different binding pose for sulfonamides. These discrepancies highlight the importance of A_2B_AR crystal structures that are urgently needed to gain reliable structural insights into the ligand-binding pocket. In fact, our new irreversible A_2B_AR antagonists may facilitate the stabilization of the A_2B_AR for structural studies.

## 4. Materials and Methods

### 4.1. Chemistry

#### 4.1.1. General

All of the reagents and solvents used in this study were purchased from various vendors (Merck (Darmstadt, Germany), Enamine (Riga, Latvia), BLDpharmtech GmbH, Kaiserslautern, Germany, or Acros Organics, Geel, Belgium, respectively) and used without purification or drying. The reactions were monitored using thin-layer chromatography (TLC). Column chromatography of the products was performed with a CombiFlash *R_f_* Companion System using RediSep packed columns. ^1^H- and ^13^C-NMR data were collected on a Bruker Avance 500 MHz NMR spectrometer (Bruker Corporation, Billerica, MA, USA)at 500 MHz (^1^H), and 126 MHz (^13^C), or on a Bruker Avance 600 MHz NMR spectrometer (Bruker, Karlsruhe, Germany) at 600 MHz (^1^H), and 151 MHz (^13^C). DMSO-d_6_ was used as solvent. Chemical shifts are reported in parts per million (ppm) relative to the peaks observed for DMSO-d_6_, ^1^H: 2.50 ppm; ^13^C: 39.5 ppm. Coupling constants *J* are reported in Hertz, and spin multiplicities are given as s (singlet), d (doublet), t (triplet), m (multiplet) and br (broad).

The purity of all compounds was determined by HPLC-UV using an LC-MS instrument (Applied Biosystems API 2000 LC-MS/MS, HPLC Agilent 1100 (Thermo Fisher Scientific, Langerwehe, Germany, and Agilent Technologies, Waldbronn, Germany. Compounds (0.5 mg/mL) were dissolved in methanol/H_2_O (1:1). Chromatographic separation was performed on a Phenomenex Luna C18 HPLC column (Phenomenex, Aschaffenburg, Germany) (50 mm × 2.00 mm, particle size 3 µm). Sample solution (10 µL) was injected into the column, and subsequently chromatographed using a gradient of water/methanol (containing 2 mM ammonium acetate) from 90%:10% to 0%:100%. Run time was set at 20 min at a flow rate of 0.25 mL/min. Mass spectra were recorded using an API 2000 mass spectrometer (electron spray ion source) coupled with an Agilent 1100 HPLC system. UV spectra were determined from 200 to 950 nm using a diode array detector. Starting compounds (**4**, **7**, **8**) were obtained from commercial sources. Detailed synthetic procedures for the required building blocks (**2a**–**c**, **3a**–**c**, **5a**–**c**, **9**–**11**) are described in Supplementary Materials; they were obtained in analogy to previously described methods [18,19,20,22,30,31,33,42,43] with some modifications.

#### 4.1.2. General Procedure A

This procedure was applied for the preparation of **2a**–**c**. To a solution of the appropriate 6-amino-3-propyluracil derivative (**1a**–**c**, 5.0 mmol) dissolved in 15 mL of acetic acid (50% aq.) and heated at 60 °C, sodium nitrite (15.0 mmol) was added portion-wise and the reaction mixture was heated for further 10 min. Upon completion of the reaction, the reaction mixture was cooled on an ice bath, and 30 mL of distilled water was added and the formed precipitate was collected by filtration. The obtained product was used in the next step without further purification.

#### 4.1.3. General Procedure B

This procedure was applied for the preparation of **3a**–**c**. To a solution of uracil derivatives (**2a**–**c**, 6.0 mmol) dissolved in 15 mL of aqueous ammonium hydroxide solution (12.5% aq.) and heated at 60 °C until the solid is completely soluble, Na_2_S_2_O_4_ (12.0 mmol) was added portion-wise till the yellow or violet color disappears forming a colorless solution. The reaction mixture was cooled on an ice bath, and 30 mL of distilled water was added, and the formed precipitate was collected by filtration. The obtained product is chemically unstable, it was therefore used directly without further purification.

#### 4.1.4. General Procedure C

This procedure was applied for the preparation of **5a**–**c** and **10**. To a flask containing the appropriate uracil derivative (**3a**–**c**, 1.2 eq), and the appropriate benzoic acid derivative (**4** or **9**, 1.0 eq) dissolved in 5–10 mL of DMF, the coupling reagent EDC (1.5 eq) was added and the reaction mixture was stirred at rt for 2–4 h. Upon completion of the reaction, 10 mL of water was added and the formed precipitate was collected by filtration. The obtained precipitate was purified by column chromatography using the eluent system DCM/methanol (9.5:0.5).

#### 4.1.5. General Procedure D

This procedure was applied for the preparation of **6a**–**c** and **11**. To a flask containing the carboxamide derivative (**5a**–**c** or **10**, 1.0 mmol) 4.0 g of PPSE was added, and the mixture was heated for 10 min at 120 °C and then for 4 h at 170 °C. After cooling to rt, 20 mL of methanol were added and the formed precipitate was filtered and washed with methanol. The obtained precipitate was purified by column chromatography using the eluent system DCM/methanol (9.7:0.3).

#### 4.1.6. Chemical Analysis

**4-(2,6-Dioxo-1-propyl-2,3,6,7-tetrahydro-1*H*-purin-8-yl)benzenesulfonyl fluoride (6a).** This compound was synthesized according to general procedure D using the carboxamide derivative **5a**. A white precipitate was obtained in a yield of 63% (225 mg). R_f_ in DCM/methanol (9.7:0.2) = 0.47. M.p.: >300 °C. ^1^H NMR (600 MHz, DMSO-d_6_) δ 14.20 (s, 1H, NH xanthine), 11.99 (s, 1H, NH xanthine), 8.42 (d, J = 8.4 Hz, 2H, CH phenyl), 8.26 (d, J = 8.7 Hz, 2H, CH phenyl), 3.87–3.76 (m, 2H, CH_2_ propyl), 1.67–1.48 (m, 2H, CH_2_ propyl), 0.87 (t, J = 7.4 Hz, 3H, CH_3_ propyl).^13^C NMR (151 MHz, DMSO-d_6_) δ 155.2, 151.1, 147.7, 147.2, 136.2, 131.9 (d, J = 23.1 Hz), 129.4, 127.8, 41.7, 21, 11.3. HPLC-UV (254 nm) ESI-MS, purity: 95.3%. LC-MS positive mode (*m*/*z*): 353.1 [M + H]^+^, calcd. *m*/*z*: [M + H]^+^ for C_14_H_13_FN_4_O_4_S 353.1.

**4-(3-Methyl-2,6-dioxo-1-propyl-2,3,6,7-tetrahydro-1*H*-purin-8-yl)benzenesulfonyl fluoride (6b).** This compound was synthesized according to general procedure **D** using the carboxamide derivative **5b**. A white precipitate was obtained in a yield of 52% (192 mg). *R_f_* in DCM/methanol (9.5:0.5) = 0.35. M.p.: >300 °C. ^1^H NMR (600 MHz, DMSO-*d*_6_) δ 14.36 (s, 1H, N*H* xanthine), 8.46 (d, *J* = 8.4 Hz, 2H, C*H* phenyl), 8.27 (d, *J* = 8.7 Hz, 2H, C*H* phenyl), 3.91–3.80 (m, 2H, C*H*_2_ propyl), 3.50 (s, 3H, C*H*_3_), 1.62–1.53 (m, 2H, C*H*_2_ propyl), 0.88 (t, *J* = 7.5 Hz, 3H, C*H*_3_ propyl). ^13^C NMR (151 MHz, DMSO-*H*) δ 154.4, 151.1, 148.6, 147, 144.4, 135.9, 132.1 (d, *J* = 23.0 Hz), 129.4, 128.9, 127.8, 109.2, 42.5, 29.9 20.9, 11.3. HPLC-UV (254 nm) ESI-MS, purity: 98.7%. LC-MS positive mode (*m*/*z*): 367.1 [M + H]^+^, calcd. *m*/*z*: [M + H]^+^ for C_15_H_15_FN_4_O_4_S 367.1.

**4-(3-Cyclopropyl-2,6-dioxo-1-propyl-2,3,6,7-tetrahydro-1*H*-purin-8-yl)benzenesulfonyl fluoride (6c).** This compound was synthesized according to general procedure **D** using the carboxamide derivative **5c**. A white precipitate was obtained in a yield of 68% (265 mg). *R_f_* in DCM/methanol (9.5:0.5) = 0.65. M.p.: >300 °C. ^1^H NMR (600 MHz, DMSO-*d*_6_) δ 14.24 (s, 1H, N*H* xanthine), 8.46 (d, *J* = 8.6 Hz, 2H, C*H* phenyl), 8.27 (d, *J* = 8.6 Hz, 2H, C*H* phenyl), 3.88–3.79 (m, 2H, C*H*_2_ propyl), 3.03 (tt, *J* = 7.2, 3.9 Hz, 1H, C*H* cyclopropyl), 1.61–1.52 (m, 2H, C*H*_2_ propyl), 1.11–1.05 (m, 2H, C*H*_2_ cyclopropyl), 1.05–0.99 (m, 2H, C*H*_2_ cyclopropyl), 0.87 (t, *J* = 7.5 Hz, 3H, C*H*_3_ propyl). ^13^C NMR (151 MHz, DMSO-*d_6_*) δ 154.7, 151.5, 149, 146.6, 136.3, 131.9 (d, *J* = 23.0 Hz), 129.4, 127.8, 109.9, 42.4, 26.4, 20.9, 11.4, 7.9. HPLC-UV (254 nm) ESI-MS, purity: 99.9%. LC-MS positive mode (*m*/*z*): 393.1 [M + H]^+^, calcd. *m*/*z*: [M + H]^+^ for C_17_H_17_FN_4_O_4_S 393.1.

**4-((1-((4-(2,6-Dioxo-1-propyl-2,3,6,7-tetrahydro-1*H*-purin-8-yl)phenyl)sulfonyl)piperidin-4-yl)carbamoyl)benzenesulfonyl fluoride (12).** To a flask containing **11** (20 mg, 0.046 mmol) and **4** (12 mg, 0.055 mmol) dissolved in 2 mL of DMF, HATU (30 mg, 0.08 mmol) and DIPEA (13 µL, 0.07 mmol) were added and the reaction mixture was stirred at rt for 18 h. Upon completion of the reaction, 10 mL of water was added and the formed precipitate was collected by filtration. The obtained precipitate was purified by column chromatography using the eluent system DCM/methanol (9.8:0.2). A white precipitate was obtained in a yield of 30% (8 mg). *R_f_* in DCM/methanol (9.5:0.5) = 0.42. M.p.: >300 °C. ^1^H NMR (600 MHz, DMSO-*d*_6_) δ 14.02 (s, 1H, N*H* xanthine), 11.95 (s, 1H, N*H* xanthine), 8.68 (s, 1H, N*H*), 8.33 (d, *J* = 8.6 Hz, 2H, C*H* phenyl), 8.21 (d, *J* = 8.8 Hz, 2H, C*H* phenyl), 8.11 (d, *J* = 8.4 Hz, 2H, C*H* phenyl), 7.92–7.81 (m, 2H, C*H* phenyl), 3.86–3.79 (m, 3H, C*H* piperidyl), 3.70–3.63 (m, 2H, C*H*_2_ propyl), 2.55 (td, *J* = 12.1, 2.9 Hz, 2H, C*H* piperidyl), 1.95–1.85 (m, 2H, C*H* piperidyl), 1.65–1.59 (m, 2H, C*H*_2_ propyl), 1.59–1.55 (m, 2H, C*H* piperidyl), 0.88 (t, *J* = 7.4 Hz, 3H, C*H*_3_ propyl). ^13^C NMR (151 MHz, DMSO) δ 164.1, 155.1, 151.1, 148.2, 147.8, 141.7, 136.9 (d, *J* = 23.1 Hz), 133.7, 133, 129.3, 128.7, 128.3, 127.2, 108.7, 46.2, 45.2, 41.7, 31.4, 30.6, 29.2, 28.8, 22.2, 21, 14.1, 11.3. HPLC-UV (254 nm) ESI-MS, purity: 95.4%. LC-MS positive mode (*m*/*z*): 619.3 [M + H]^+^, calcd. *m*/*z*: [M + H]^+^ for C_26_H_27_FN_6_O_7_S_2_ 619.1.

### 4.2. Receptor Expression and Membrane Preparation

The human A_2A_AR, the mutant human A_2B_AR (K269^7.32^L), and the human A_3_AR were transiently expressed in FreeStyle^TM^ CHO-S cells using the transfection reagent polyethylenimine (PEI) at a ratio of 1:3 to DNA according to the manufacturer’s guideline. pcDNA3.1(+) was used as the transfection vector containing the cDNA for either the wt ARs or the K269^7.32^L mutant A_2B_AR that was prepared by site-directed mutagenesis [44] with the following primer: forward TCAGGGTAAAAATAAGCCCCTGTGGGCAATGAATATGGCCA, reverse TGGCCATATTCATTGCCCACAGGGGCTTATTTTTACCCTGA. CHO-S cells were grown in FreeStyle^TM^ CHO Expression Medium supplemented with 8 mM l-glutamine before use and split to 0.5 mio. cells per mL the day before transfection. Twenty-four hours after transfection, cells were spun down by centrifugation for 15 min at 500× *g*. Cells were disrupted by resuspension in 5 mM tris(hydroxymethyl)aminomethane buffer (Tris, pH 7.4) containing 2 mM ethyl-ene¬diaminetetraacetic acid (EDTA) with subsequent homogenization using an Ultra-Turrax homogenizer. The cell suspension was centrifuged for 10 min at 1000× *g* and the obtained supernatant was pelleted by centrifugation for 60 min at 48,000× *g*. The supernatant was discarded and the pellet washed with 50 mM Tris (pH 7.4). After centrifugation for at least 30 min at 48,000× *g*, the pellet was resuspended in 50 mM Tris (pH 7.4), aliquoted and stored at −80 °C for further use. All centrifugation steps were carried out at 4 °C. Membrane preparations of the human wt A_1_AR and the human wt A_2B_AR were purchased from Perkin Elmer (Perkin-Elmer, Waltham, MA, USA) (catalogue numbers ES-010-M400UA and ES-013-M400UA).

### 4.3. Radioligand Binding Experiments

Binding experiments were performed using 50 mM Tris buffer (pH 7.4) in a total volume of 400 µL for the A_1_AR, A_2A_AR, A_3_AR, and 1000 µL for the A_2B_AR. Initial screening was carried out in two independent experiments at 1 µM. Competition of compounds versus [^3^H]CCPA (A_1_AR, final concentration 1 nM), [^3^H]MSX-2 (A_2A_AR, final concentration 1 nM), [^3^H]PSB-603 (A_2B_AR, final concentration 0.3 nM) and [^3^H]PSB-11 (A_3_AR, final concentration 1 nM) was investigated. An amount of 7.5–25 µg of total protein per well was employed, incubated with 2 U of adenosine deaminase per mL beforehand. Non-specific binding was determined in the presence of 10 µM of 2-chloroadenosine, 10 µM of CGS 15943, 10 µM of 8-cyclopently-1,3-dipropylxanthine or 100 µM of (*R*)-*N*^6^-phenylisopropyadenosine for A_1_-, A_2A_-, A_2B_- and A_3_ARs, respectively. Competition association experiments (at 25 °C) were performed in the same way but the protein was added delayed at indicated time points and the cell membranes were harvested after 1 min. Incubation was terminated by rapid filtration through GF/B glass fiber filters (Whatman) using a 48-well harvester (Brandel) after 90 (A_1_AR), 30 (A_2A_AR), 75 (A_2B_AR), and 45 min (A_3_AR) of incubation, respectively. Filters were pre-incubated with 0.3% (*w*/*v*) of PEI for 30–60 min in case of the A_2A_AR assay. Filters were rinsed three times with ice-cold 50 mM Tris, pH 7.4 (supplemented with 0.1% (*w*/*v*) bovine serum albumin for the A_2B_AR) and transferred into scintillation vials. Filters were then incubated with 2.5 mL of scintillation cocktail (ProSafe FC plus) for at least 6 h. Subsequently, radioactivity was determined using a liquid scintillation counter (Tricarb 2810TR, Perkin Elmer). Data were analyzed using GraphPad Prism 7.0 (San Diego, CA, USA. K_i_ values of competition binding studies were calculated using the implemented equations for one-site fit; the K_i_ values were calculated from IC_50_ values by the Cheng-Prusoff equation. The K_D_ value at the mutant receptor was determined by homologous competition binding of PSB-603 vs. [^3^H]PSB-603 and calculated by the one site-homologous fit equation in Prism. Control and competitive association data of kinetic experiments were fitted to the implemented one-phase association, and kinetics of competitive binding using previously obtained parameters [22].

### 4.4. Wash-Out Experiments

A_2B_AR membrane preparations were incubated with a concentration representing 100-fold of the K_i_ value for reversible ligands and 10-fold of the K_i_ value for irreversible ligands. After 2 h of incubation at rt, mixtures were divided up into two batches. One of them was kept on ice and the other one was centrifuged for 10 min at 20,627× *g* and 4 °C. The pellet was resuspended in 50 mM Tris buffer (pH 7.4) and centrifuged again for 10 min at 20,627× *g* and 4 °C. The described washing procedure was repeated four times. The final pellet was resuspended in assay buffer and subjected to the A_2B_AR binding assay together with the batch stored on ice to determine radioligand binding capacity. This procedure resulted in a 10-times dilution of the membrane samples in the final assay volume. Control samples that were incubated with DMSO instead of an antagonist were treated in the same way and were used to determine total and non-specific binding. The determined radioligand binding of the samples was then normalized to the controls.

### 4.5. Functional G Protein Activation Experiments Using BRET² Assays

Inhibition of NECA-induced G protein activation by xanthine derivatives was monitored by means of the TRUPATH BRET² assay with a slightly modified protocol [39].

HEK293 cells were seeded into 6-well plates at a density of 5 × 10^5^ cells per well and incubated in Dulbecco’s Modified Eagle’s Medium (DMEM), 10% fetal calf serum (FCS), and 0.1 mg mL^−1^ streptomycin + 100 U/mL penicillin overnight at 37 °C and 5% CO_2_. After 2 h, the media was removed and replaced with 2 mL of serum-free OptiMEM (Gibco, ThermoFisher, Waltham, MA, USA) per well. Transient transfection was performed with 2.5 µL of lipofectamine 2000 (ThermoFisher) as a transfection reagent, and each 100 ng of pcDNA5/FRT/TO-Gα_15_-RLuc8, pcDNA3.1-Gβ_3_, pcDNA-Gγ_13_-GFP2, and pcDNA3.1-ADORA2B per well. Lipofectamine was diluted to 250 µL with OptiMEM and mixed for 20 min before it was added to the DNA mixture (diluted to 250 µL in OptiMEM as well). The mixture was incubated for 30 min at rt and added to the cells overnight. On the next day, the transfection mixture was aspirated from the cells and replaced with 2 mL of DMEM. Twenty-four hours after transfection, cells were detached using phosphate-buffered saline (PBS) and 0.5 mM EDTA. Cells were seeded into white 96-well plates at a density of 30,000 cells per well in a total volume of 100 µL DMEM.

The next day, the media was removed, and the cells were washed once with assay buffer (Hank’s balanced salt solution + 20 mM 2-[4-(2-hydroxyethyl)piperazin-1-yl]ethane-1-sulfonic acid [HEPES], pH 7.4) and incubated with 70 µL of assay buffer. Antagonist solution (10 µL) was added to the cells and incubated for 1h if not indicated otherwise. In washout experiments, the cells were washed twice with 100 µL of assay buffer after antagonist incubation. Subsequently, 10 µL of substrate solution (50 µM Deep Blue C, Biomol, Hamburg, Germany) was added to the cells and the mixture was incubated for 5 min before addition of agonist (10 µL of NECA at its EC_80_ concentration, 100 nM final concentration, as previously determined [38]). RLuc8-luminescence (395 nm emission filter) and GFP2-fluorescence (515 nm emission filter) were measured 5 min after agonist addition using a Mithras LB940 plate reader. The BRET^2^ ratio (GFP2 counts divided by RLuc8 counts) was baseline-corrected using BRET^2^ ratios from samples without either agonist or antagonist to obtain ΔBRET, also known as agonist-promoted BRET. ΔBRET values were plotted with GraphPad Prism 8.0 and fitted to a three-parameter sigmoidal equation to obtain IC_50_ values of the antagonists.

### 4.6. Docking Experiments

Covalent docking of PSB-21500 (**6a**) was performed with the covalent docking option in the GLIDE docking module implemented in the Maestro suite (Schrödinger, New York, NY, USA). A previously published homology model of the A_2B_AR was used [18]. The reaction type was set to nucleophilic substitution, the reactive atom to the fluorine atom in the ligand, and the reagent residue to K269. Remaining options remained at default settings. Five docking poses were manually evaluated regarding their plausibility. The docking result figure was generated using PyMOL (Schrödinger).

## Figures and Tables

**Table 1:** (Apparent) affinities of new xanthine derivatives at human adenosine receptors ^1^.

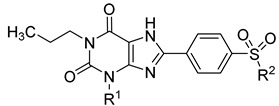			A_1_AR vs. Radioligand [^3^H]CCPA	A_2A_AR vs. Radioligand [^3^H]MSX-2	A_2B_AR vs. Radioligand [^3^H]PSB-603	A_3_AR vs. Radioligand [^3^H]PSB-11
**Name**	**R^1^**	**R^2^**	**K_i_ ± SEM (nM)**, (or % inhibition at 1 µM)			
PSB-1115 (**IV**)	H	OH	**>10,000 ^2^**	**3790** ± 520 ^3^	**152** ± 12 [**53.4**] ^4^	**>10,000 ^2^**
**6a** PSB-21500	H	F	**406** ± 194	**~1000** (53%)	**10.6** ± 0.9	**>1000** (27%)
**6b** PSB-21501	methyl	F	**293** ± 92	**372** ± 48	**15.9** ± 1.0	**>1000** (13%)
**6c** PSB-21502	cyclo- propyl	F	**41.5** ± 2.8	**72.9** ± 8.1	**26.0** ± 2.8	**>1000** (33%)
**12** PSB-21503	H	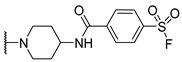	**>1000** (22%)	**>1000** (32%)	**7.37** ± 0.83	**>1000** (15%)

^1^ K_i_ values were determined as means from three independent experiments shown in bold ± standard error of the mean (SEM). K_i_ values represent apparent K_i_ values for irreversibly binding ligands, determined as described in the Section 4. ^2^ Reference [22]. ^3^ Reference [37]. ^4^ The previously published K_i_ value using the radioligand [^3^H]ZM241385 [19].

## Data Availability

All data are contained within this article or its Supplementary Materials or are available upon reasonable request.

## Supplementary Materials

The following supporting information can be downloaded at: https://www.mdpi.com/article/10.3390/molecules27123792/s1. Preparation of key intermediates, and NMR and LC-MS data for final compounds.

## Sample Availability

Samples of the compounds are available upon reasonable request and appropriate compensation, depending on availability.
