# Supplementary material for: Irreversible Antagonists for the Adenosine A2B Receptor
Source: Molecules. 2022 Jun 13;27(12):3792. doi: 10.3390/molecules27123792 (PMC9231011; doi:10.3390/molecules27123792)
Supplement: Supplementary file 1 [file molecules-27-03792-s001.zip › molecules-1760172-supplementary.pdf]

Supporting Information

Irreversible antagonists for the adenosine A_{2B} receptor

Ahmed Temirak,[1&] Jonathan G. Schlegel,[1&] Jan H. Voss,[1] Victoria J. Vaaßen,[1] Christin Vielmuth,[1] Tobias Claff,[1] and Christa E. Müller [1,*]

[1]PharmaCenter Bonn, Pharmaceutical Institute, Department of Pharmaceutical & Medicinal Chemistry, University of Bonn, An der Immenburg 4, D-53121 Bonn, Germany

[&]Equal contribution

*Correspondence: christa.mueller@uni-bonn.de

Table of contents

Preparation of key intermediates **2a**–**c**, **3a**–**c**, **5a**–**c**, **9**–**11**……..………………………. S2–S6

NMR data for compounds **6a**–**c** and **12**………………………….…...…….……...... S7–S10

LC/MS data for compounds **6a**–**c** and **12**.……………………………………..……… S11–S18

6-Amino-5-nitroso-3-propyl-1*H*-pyrimidine-2,4-dione (2a) [30]. This compound was synthesized according to general procedure **A**. A yellow precipitate was obtained in a yield of 90% (890 mg). ^{1}H NMR (500 MHz, DMSO-d_6) δ 11.37 (s, 1H, N*H* uracil), 3.81-3.75 (t, *J* = 6.2 Hz, 2H, CH_2 propyl), 1.63-1.54 (m, 2H, CH_2 propyl), 0.88 (t, *J* = 7.5 Hz, 3H, CH_3 propyl). ^{13}C NMR (126 MHz, DMSO-d_6) δ 161.7, 150.8, 146.3, 140.1, 41.4, 21.0, 11.3. HPLC-UV (254 nm) ESI-MS, purity: 97.5%. LC-MS positive mode (m/z): 199.2 $[M + H]^+$.

6-Amino-1-methyl-5-nitroso-3-propylpyrimidine-2,4(1*H*,3*H*)-dione (2b) [43]. This compound was synthesized according to general procedure **A**. A violet precipitate was obtained in a yield of 94% (990 mg). ^{1}H NMR (500 MHz, DMSO-d_6) δ 3.88-3.82 (m, 2H, CH_2 propyl), 3.23 (d, *J* = 25.8 Hz, 3H, CH_3), 1.66-1.55 (m, 2H, CH_2 propyl), 0.89 (t, *J* = 7.4 Hz, 3H, CH_3 propyl). ^{13}C NMR (126 MHz, DMSO-d_6) δ: 160.1, 149.3, 146.3, 139.3, 42.5, 28.7, 20.8, 11.3. HPLC-UV (254 nm) ESI-MS, purity: 99.1%. LC-MS positive mode (m/z): 212.1 $[M + H]^+$.

6-Amino-1-cyclopropyl-5-nitroso-3-propylpyrimidine-2,4(1*H*,3*H*)-dione (2c). This compound was synthesized according to general procedure **A**. A violet precipitate was obtained in a yield of 81% (965 mg). ^{1}H NMR (600 MHz, DMSO-d_6) δ 3.84-3.77 (t, *J* = 7.4 Hz, 2H, CH_2 propyl), 2.61 (tt, *J* = 7.0, 3.9 Hz, 1H, C*H* cyclopropyl), 1.63-1.53 (m, 2H, CH_2 propyl), 1.17-1.10 (m, 2H, CH_2 cyclopropyl), 0.88 (t, *J* = 7.5 Hz, 3H, CH_3 propyl), 0.81-0.77 (m, 2H, CH_2 cyclopropyl). ^{13}C NMR (151 MHz, DMSO-d_6) δ: 172.1, 160.5, 149.4, 147.4, 139.7, 42.4, 24.4, 20.9, 11.4, 9.5. HPLC-UV (254 nm) ESI-MS, purity: 98.6%. LC-MS positive mode (m/z): 238.8 $[M + H]^+$.

5,6-Diamino-3-propyl-1*H*-pyrimidine-2,4-dione (3a) [30]. This compound was synthesized according to general procedure **B**. A white precipitate was obtained in a yield of 68% (750 mg). ^{1}H NMR (500 MHz, DMSO-d_6) δ 12.54 (s, 1H, N*H* uracil), 5.55 (s, 2H, NH_2 uracil), 3.67-3.61 (t, *J* = 6.4 Hz, 2H, CH_2 propyl), 1.54-1.45 (m, 2H, CH_2 propyl), 0.82 (t, *J* = 7.6 Hz, 3H, CH_3

propyl). ^{13}C NMR (126 MHz, DMSO-d_6) δ 160.4, 151, 149.2, 142.4, 41, 21, 11.4. HPLC-UV (254 nm) ESI-MS, purity: 95.1%. LC-MS positive mode (m/z): 185.2 $[M + H]^+$.

5,6-Diamino-1-methyl-3-propylpyrimidine-2,4(1*H*,3*H*)-dione (3b) [43]. This compound was synthesized according to general procedure **B**. A white precipitate was obtained in a yield of 77% (918 mg). HPLC-UV (254 nm) ESI-MS, purity: 97.4%. LC-MS positive mode (m/z): 198.2 $[M + H]^+$.

5,6-Diamino-1-cyclopropyl-3-propylpyrimidine-2,4(1*H*,3*H*)-dione (3c). This compound was synthesized according to general procedure **B**. A white precipitate was obtained in a yield of 80% (1.07 g). ^1H NMR (500 MHz, DMSO-d_6) δ 6.02 (s, 2H, NH_2 xanthine), 3.67 (dd, J = 8.2, 6.6 Hz, 2H, CH_2 propyl), 2.62 (tt, J = 6.9, 3.8 Hz, 1H, C*H* cyclopropyl), 1.46 (q, J = 7.6 Hz, 2H, CH_2 propyl), 1.14-1.04 (m, 2H, CH_2 cyclopropyl), 0.80 (t, J = 7.4 Hz, 3H, CH_3 propyl), 0.74-0.67 (m, 2H, CH_2 cyclopropyl). ^{13}C NMR (126 MHz, DMSO-d_6) δ: 159.2, 150.1, 145.6, 95.7, 92, 41.8, 25.2, 21, 11.3, 9.9. HPLC-UV (254 nm) ESI-MS, purity: 95.4%. LC-MS positive mode (m/z): 224.1 $[M + H]^+$.

4-((6-Amino-2,4-dioxo-3-propyl-1,2,3,4-tetrahydropyrimidin-5-yl)carbamoyl)benzenesulfonyl fluoride (5a). This compound was synthesized according to general procedure **C** using **3a** (1.09 g, 5.9 mmol) and **4** (1.0 g, 4.9 mmol) dissolved in 10 mL DMF, EDC (1.4 g, 7.4 mmol) was added and the reaction mixture was stirred at rt for 2 h. A pale-yellow precipitate was obtained in a yield of 66% (75 mg). R_f in DCM/methanol (9.5:0.5) = 0.43. ^1H NMR (500 MHz, DMSO-d_6) δ 10.71 (s, 1H, N*H* uracil), 9.27 (s, 1H, N*H* uracil), 8.26 (d, J = 3.2 Hz, 4H, C*H* phenyl), 6.44 (s, 2H, NH_2), 3.70-3.58 (m, 2H, CH_2 propyl), 1.59-1.41 (m, 2H, CH_2 propyl), 0.83 (t, J = 7.5 Hz, 3H, CH_3 propyl). ^{13}C NMR (126 MHz, DMSO-d_6) δ 164.9, 162.5, 160.6, 150.9, 149.9, 141.9, 133.6 (d, J = 23.4 Hz), 129.9, 128.4, 86.3, 35.9, 21.1, 11.3. HPLC-UV (254 nm) ESI-MS, purity: 95.1%. LC-MS positive mode (m/z): 371.2 $[M + H]^+$.

4-((6-Amino-1-methyl-2,4-dioxo-3-propyl-1,2,3,4-tetrahydropyrimidin-5-yl)carbamoyl)benzene-sulfonyl fluoride (5b). This compound was synthesized according to general procedure **C** using **3b** (116 mg, 0.59 mmol) and **4** (100 mg, 0.49 mmol) dissolved in 5 mL DMF, EDC (140 mg, 0.74 mmol) was added and the reaction mixture was stirred at rt for 2 h. A pale-yellow precipitate was obtained in a yield of 73% (136 mg). R_f in DCM/methanol (9.5:0.5) = 0.65. ^{1}H NMR (600 MHz, DMSO-d_6) δ 9.32 (s, 1H, N*H* uracil), 8.37-8.20 (m, 4H, C*H* phenyl), 6.82 (s, 2H, NH_2), 3.77-3.68 (m, 2H, CH_2 propyl), 3.33 (s, 3H, CH_3), 1.56-1.46 (m, 2H, CH_2 propyl), 0.88 (t, J = 7.4 Hz, 3H, CH_3 propyl). ^{13}C NMR (151 MHz, DMSO-d_6) δ 165.1, 159.2, 152.5, 142, 133.5 (d, J = 23.4 Hz), 130, 128.4, 127.7, 125.2, 87, 62.7, 42.1, 30.1, 21, 11.4. HPLC-UV (254 nm) ESI-MS, purity: 95.8%. LC-MS positive mode (m/z): 385.1 $[M + H]^+$.

4-((6-Amino-1-cyclopropyl-2,4-dioxo-3-propyl-1,2,3,4-tetrahydropyrimidin-5-yl)carbamoyl)-benzenesulfonyl fluoride (5c). This compound was synthesized according to general procedure **C** using **3c** (133 mg, 0.59 mmol) and **4** (100 mg, 0.49 mmol) dissolved in 5 mL DMF, EDC (140 mg, 0.74 mmol) was added and the reaction mixture was stirred at rt for 4 h. A pale-yellow precipitate was obtained in a yield of 70% (136 mg). R_f in DCM/methanol (9.8:0.2) = 0.36. ^{1}H NMR (600 MHz, DMSO-d_6) δ 9.26 (s, 1H, N*H* uracil), 8.31-8.21 (m, 4H, C*H* phenyl), 6.79 (s, 2H, NH_2), 3.71-3.65 (m, 2H, CH_2 propyl), 2.68 (tt, J = 7.0, 3.9 Hz, 1H, C*H* cyclopropyl), 1.52-1.45 (m, 2H, CH_2 propyl), 1.17 (dt, J = 7.9, 3.8 Hz, 2H, CH_2 cyclopropyl), 0.82 (t, J = 7.4 Hz, 3H, CH_3 propyl), 0.78-0.73 (m, 2H, CH_2 cyclopropyl). ^{13}C NMR (151 MHz, DMSO-d_6) δ 165, 159.4, 153.1, 150.8, 142, 133.6, (d, J = 23.4 Hz), 131.1, 130, 128.4, 86.7, 41.9, 25.5, 21.1, 11.4, 10.2. HPLC-UV (254 nm) ESI-MS, purity: 97.7%. LC-MS positive mode (m/z): 411.2 $[M + H]^+$.

4-((4-((*tert*-Butoxycarbonyl)amino)piperidin-1-yl)sulfonyl)benzoic acid (9) [33]. To a flask containing 4-(chlorosulfonyl)benzoic acid **7** (1.0 g, 4.53 mmol) and *tert*-butyl piperidin-4-ylcarbamate **8** (998 mg, 4.53 mmol) dissolved in 10 mL of dioxane, TEA (1.26 mL, 9.06 mmol)

was added and the reaction mixture was stirred at rt for 36 h. Upon completion of the reaction, solvents were vaporized, 10 mL of distilled water was added and the formed precipitate was collected by filtration. The obtained precipitate was purified by column chromatography using the eluent system petroleum ether/ethyl acetate (7.0:3.0). A white precipitate was obtained in a yield of 70% (1.23 g). R_f in petroleum ether/ethyl acetate (7.0:3.0) = 0.32. ^{1}H NMR (500 MHz, DMSO-d_6) δ 8.13 (d, J = 8.2 Hz, 2H, CH phenyl), 7.82 (d, J = 8.2 Hz, 2H, CH phenyl), 3.48 (dt, J = 12.9, 4.4 Hz, 3H, CH piperidyl), 3.03 (q, J = 7.4 Hz, 1H, CH piperidyl), 1.81-1.68 (m, 3H, CH piperidyl), 1.34 (s, 9H, Boc), 1.19 (t, J = 7.3 Hz, 2H, CH piperidyl). ^{13}C NMR (126 MHz, DMSO) δ 167.6, 166.5, 154.9, 140.6, 130.3, 127.8, 127.7, 77.8, 46, 44.7, 30.9, 28.4, 28.4, 8.6. HPLC-UV (254 nm) ESI-MS, purity: 95.4%. LC-MS positive mode (m/z): 383.1 $[M - H]^-$.

***tert*-Butyl (1-((4-((6-amino-2,4-dioxo-3-propyl-1,2,3,4-tetrahydropyrimidin-5-yl)carbamoyl) phenyl)sulfonyl)piperidin-4-yl)carbamate (10).** This compound was synthesized according to general procedure **C** using **3a** (574 mg, 3.12 mmol) and **9** (1.0 g, 2.6 mmol) dissolved in 5 mL DMF, EDC (747 mg, 4.0 mmol) was added and the reaction mixture was stirred at rt for 4 h. A white precipitate was obtained in a yield of 68% (980 mg). R_f in DCM/methanol (9.8:0.2) = 0.69. ^{1}H NMR (500 MHz, DMSO-d_6) δ 13.95 (s, 1H, NH uracil), 10.45 (s, 1H, NH uracil), 8.22-8.13 (m, 2H, CH phenyl), 7.79 (m, 4H, CH phenyl), 7.58 (m, 2H, CH phenyl), 6.84 (s, 2H, NH_2), 3.90 (s, 1H, CH piperidyl), 3.72-3.60 (m, 2H, CH_2 propyl), 3.50 (td, J = 8.7, 4.4 Hz, 4H, CH piperidyl), 1.82-1.70 (m, 4H, CH piperidyl), 1.56-1.46 (m, 2H, CH_2 propyl), 1.44-1.37 (t, 9H, Boc), 0.83 (t, J = 7.4 Hz, 3H, CH_3 propyl). ^{13}C NMR (126 MHz, DMSO-d_6) δ 167.6, 165.4, 160.7, 154.9, 150.7, 150.1, 140.6, 138.7, 136.7, 130.3, 129, 127.9, 127.7, 127.2, 86.7, 77.8, 46.2, 44.9, 41.1, 30.9, 28.4, 28.4, 21.1, 11.3. HPLC-UV (254 nm) ESI-MS, purity: 96%. LC-MS positive mode (m/z): 551.4 $[M + H]^+$.

***tert*-butyl (1-((4-(2,6-Dioxo-1-propyl-2,3,6,7-tetrahydro-1*H*-purin-8-yl)phenyl)sulfonyl)piperidin-4-yl)carbamate (11).** This compound was synthesized according to general procedure **D** using the carboxamide derivative **10**. A white precipitate was obtained in a yield of 60% (255 mg). R_f in DCM/methanol (9.0:1.0) = 0.26. ^{1}H NMR (500 MHz, DMSO-d_6) δ 13.95 (s, 1H, N*H* xanthine), 11.9 (s, 1H, N*H* xanthine), 8.37-8.26 (m, 2H, C*H* phenyl), 7.83 (d, *J* = 8.5 Hz, 2H, C*H* phenyl), 6.26 (s, 2H, NH_2), 3.86-3.78 (m, 3H, C*H* piperidyl), 3.70-3.61 (m, 2H, CH_2 propyl), 3.02 (m, 2H, C*H* piperidyl), 2.02-1.94 (m, 2H, C*H* piperidyl), 1.61-1.56 (m, 2H, CH_2 propyl), 1.56-1.49 (m, 2H, C*H* piperidyl), 0.87 (t, *J* = 7.4 Hz, 3H, CH_3 propyl). ^{13}C NMR (126 MHz, DMSO-d_6) δ 155.7, 155.5, 151.1, 147.6, 147, 136.3, 133.7, 128.2, 126.9, 46.4, 44.3, 41.6, 34.3, 29.1, 21, 11.3. HPLC-UV (254 nm) ESI-MS, purity: 96.3%. LC-MS positive mode (m/z): 433.3 $[M + H]^+$.

^{1}HNMR

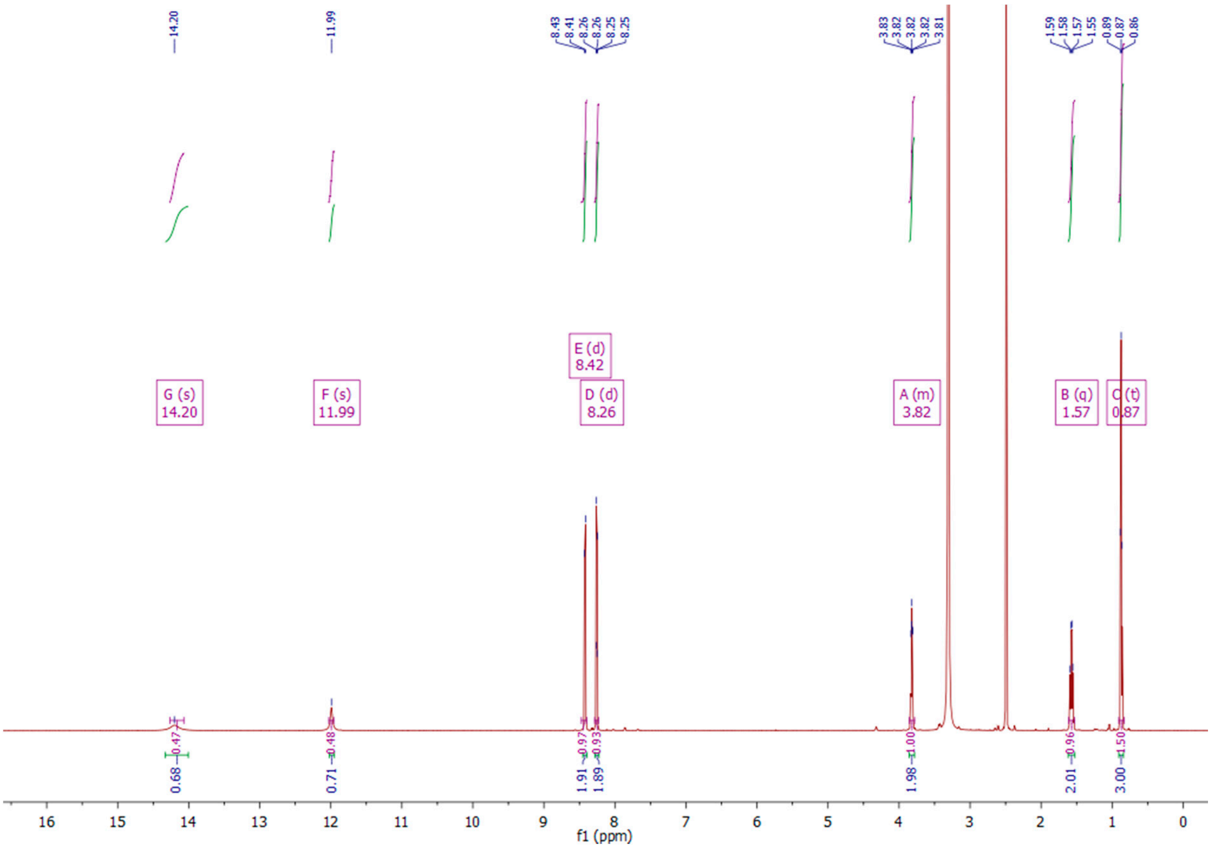

^{13}CNMR

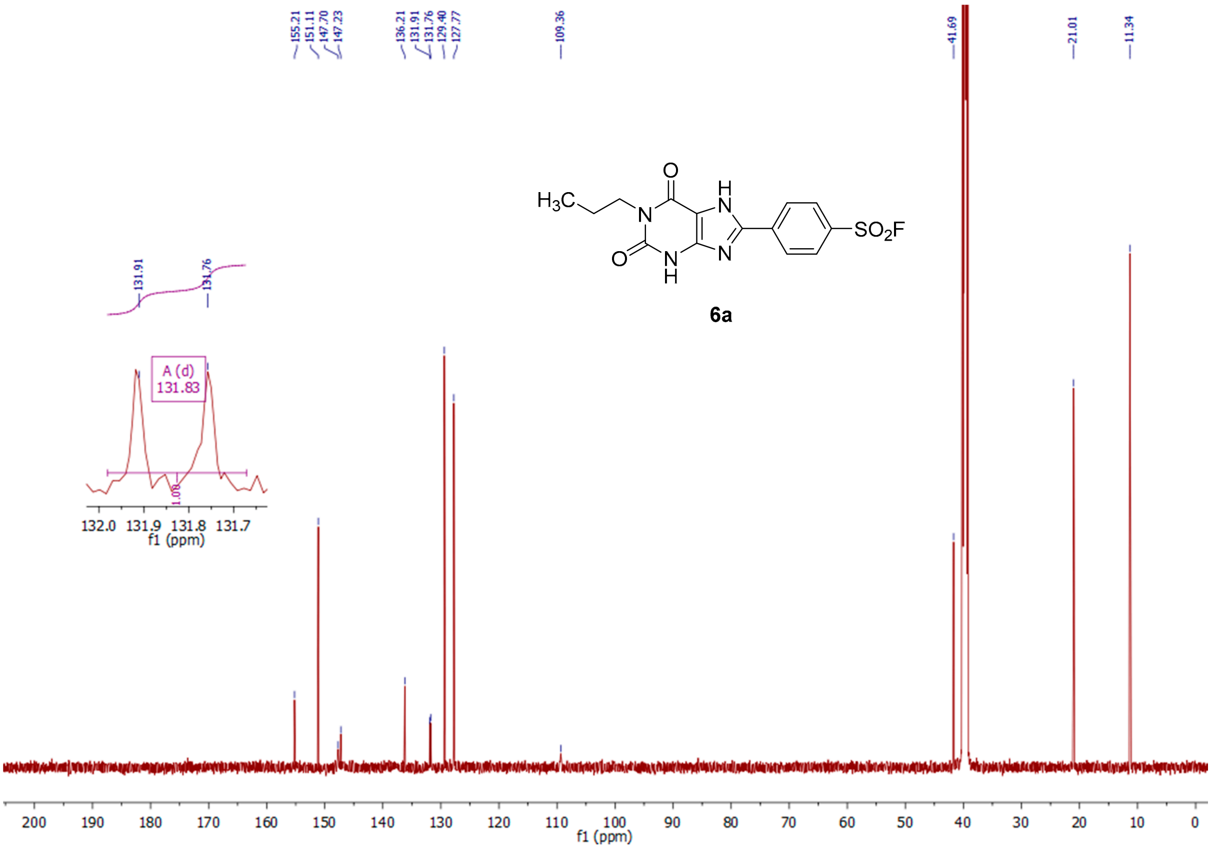

Figure S1. ^{1}H (600 MHz) and ^{13}C (151 MHz) spectra (DMSO-d_6) of 4-(2,6-dioxo-1-propyl-2,3,6,7-tetrahydro-1*H*-purin-8-yl)benzenesulfonyl fluoride (**6a**).

^{1}HNMR

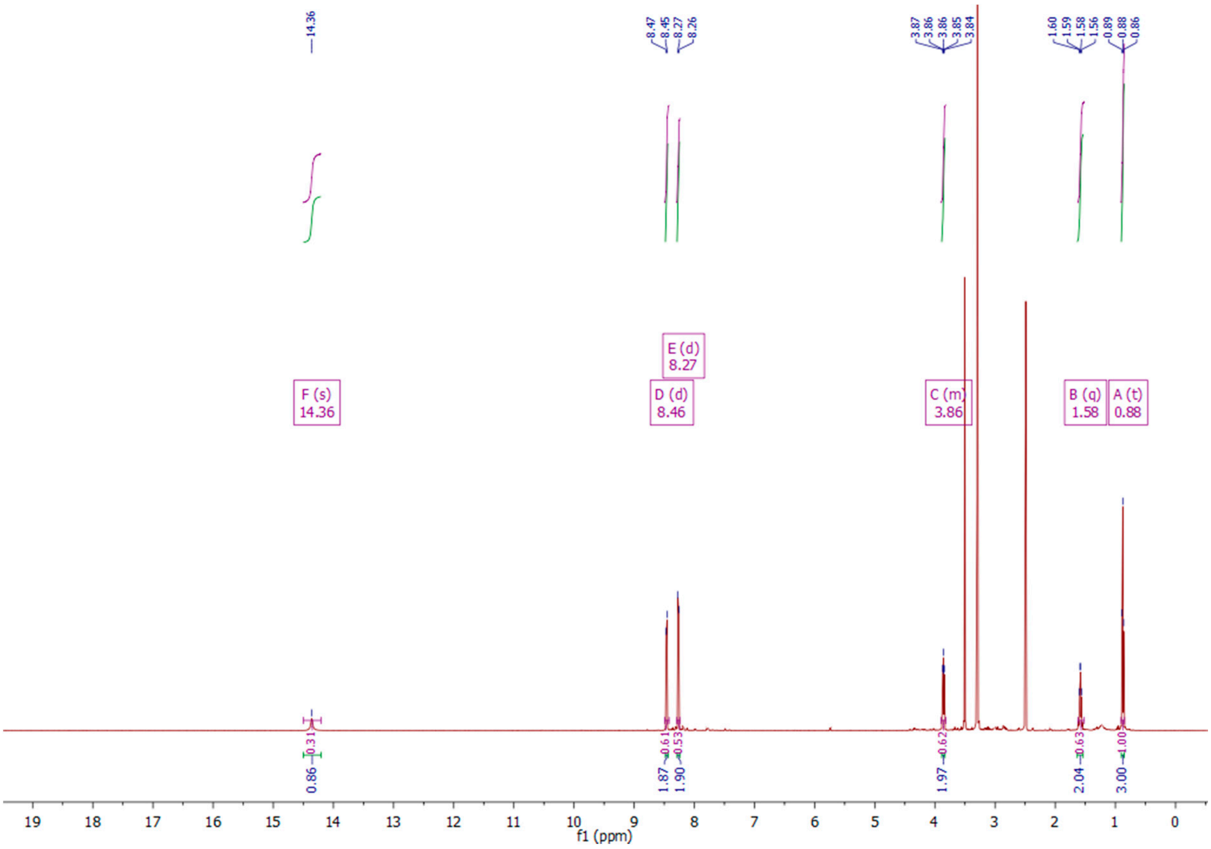

^{13}CNMR

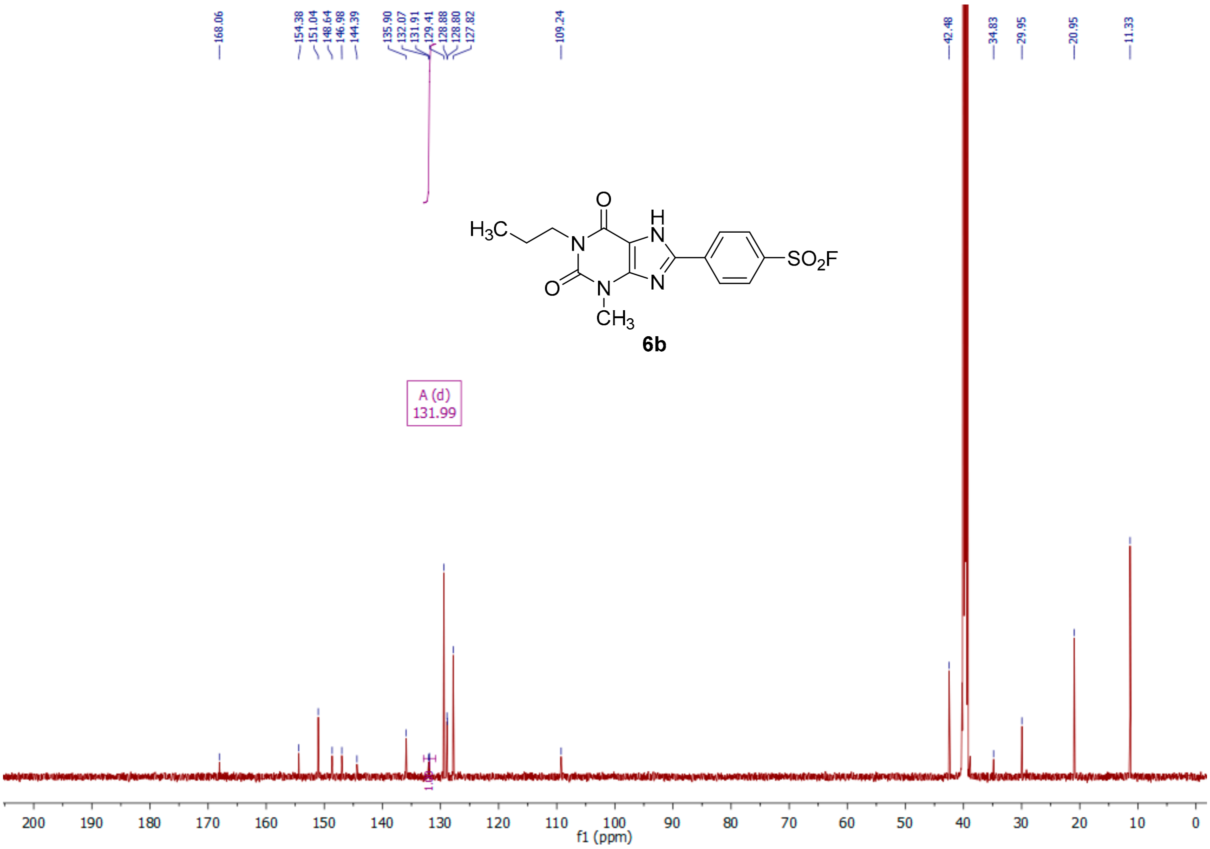

Figure S2. ^{1}H (600 MHz) and ^{13}C (151 MHz) spectra (DMSO-d_6) of 4-(3-methyl-2,6-dioxo-1-propyl-2,3,6,7-tetrahydro-1*H*-purin-8-yl)benzenesulfonyl fluoride (**6b**).

^{1}HNMR

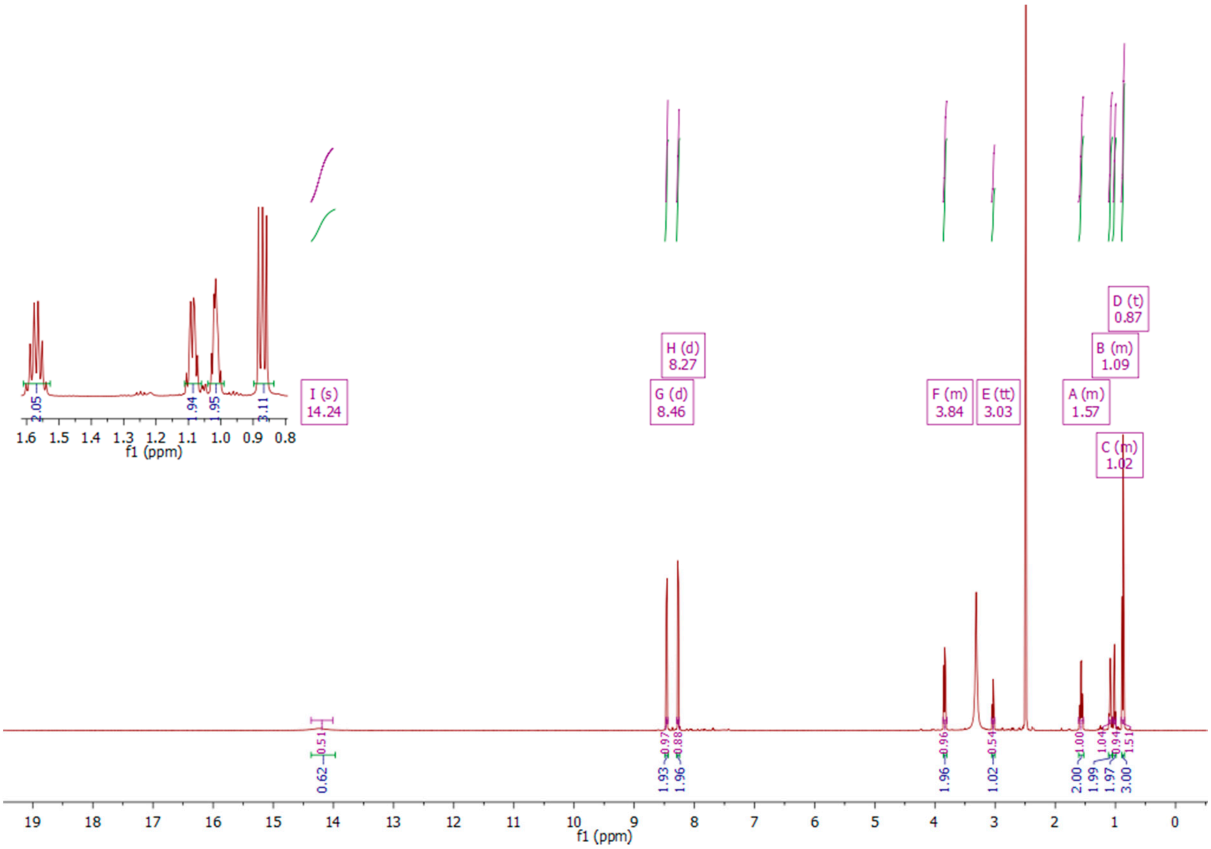

^{13}CNMR

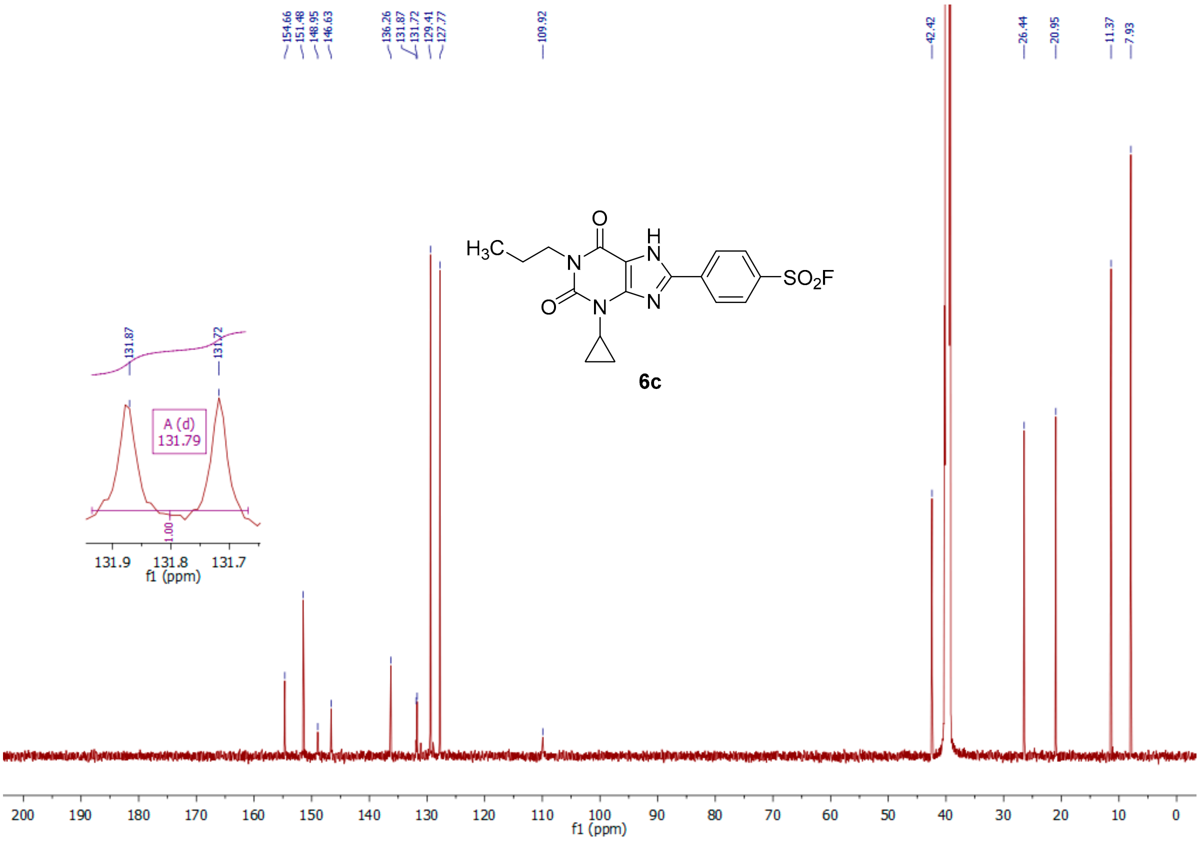

Figure S3. ^{1}H (600 MHz) and ^{13}C (151 MHz) spectra (DMSO-d_6) of 4-(3-cyclopropyl-2,6-dioxo-1-propyl-2,3,6,7-tetrahydro-1*H*-purin-8-yl)benzenesulfonyl fluoride (**6c**).

^{1}HNMR

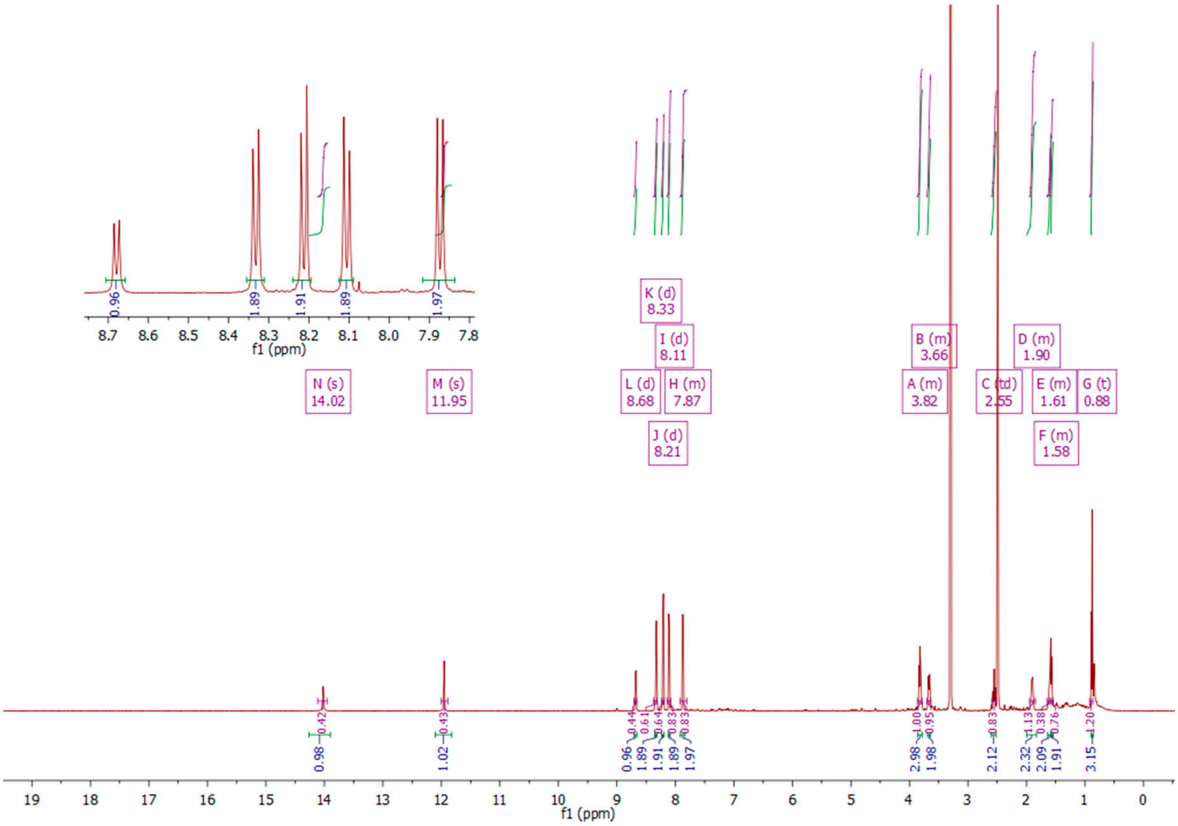

^{13}CNMR

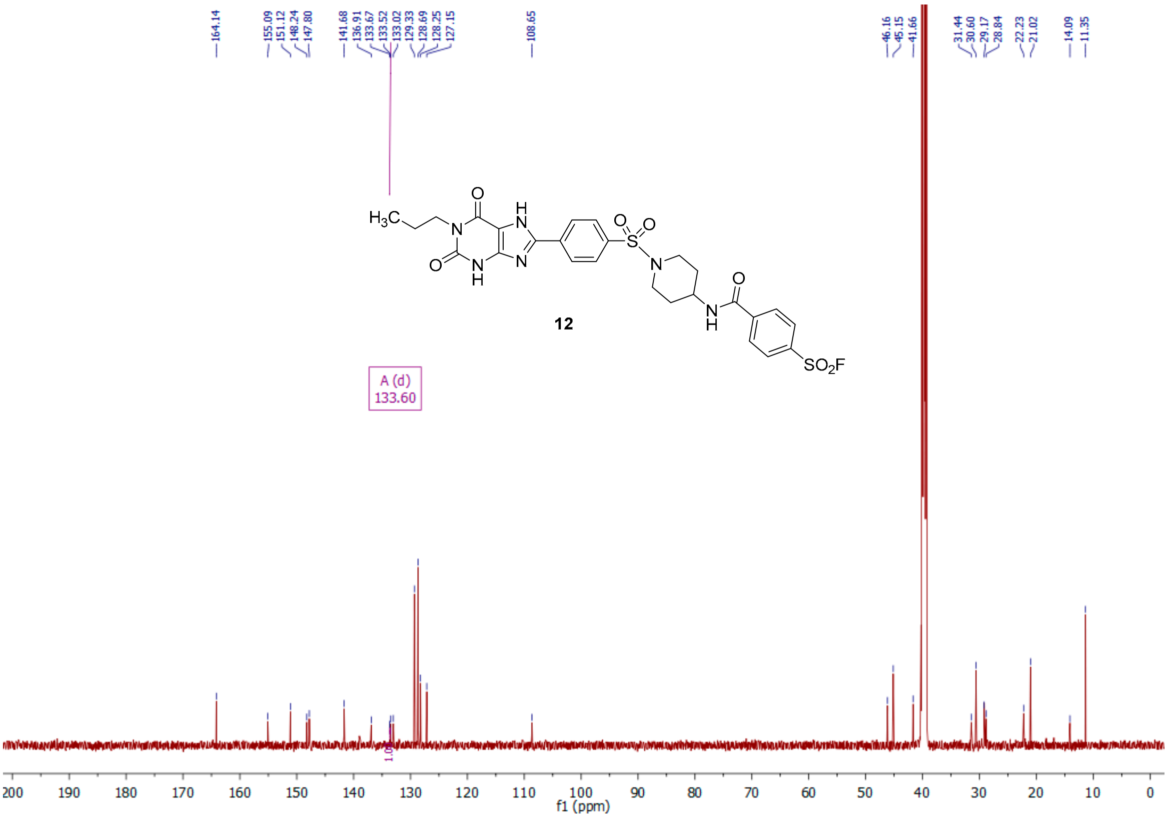

Figure S4. ^{1}H (600 MHz) and ^{13}C (151 MHz) spectra (DMSO-d_6) of 4-((1-((4-(2,6-dioxo-1-propyl-2,3,6,7-tetrahydro-1*H*-purin-8-yl)phenyl)sulfonyl)piperidin-4-yl)carbamoyl)benzene-sulfonyl fluoride (**12**).

**Inj. volume:**	5.000	**Location:**	P1-F4
**Acq. method:**	0_wasser-acn_standard_100-1000_AC-20min.amx		
**Processing method:**	MS_standard_plot.pmx		

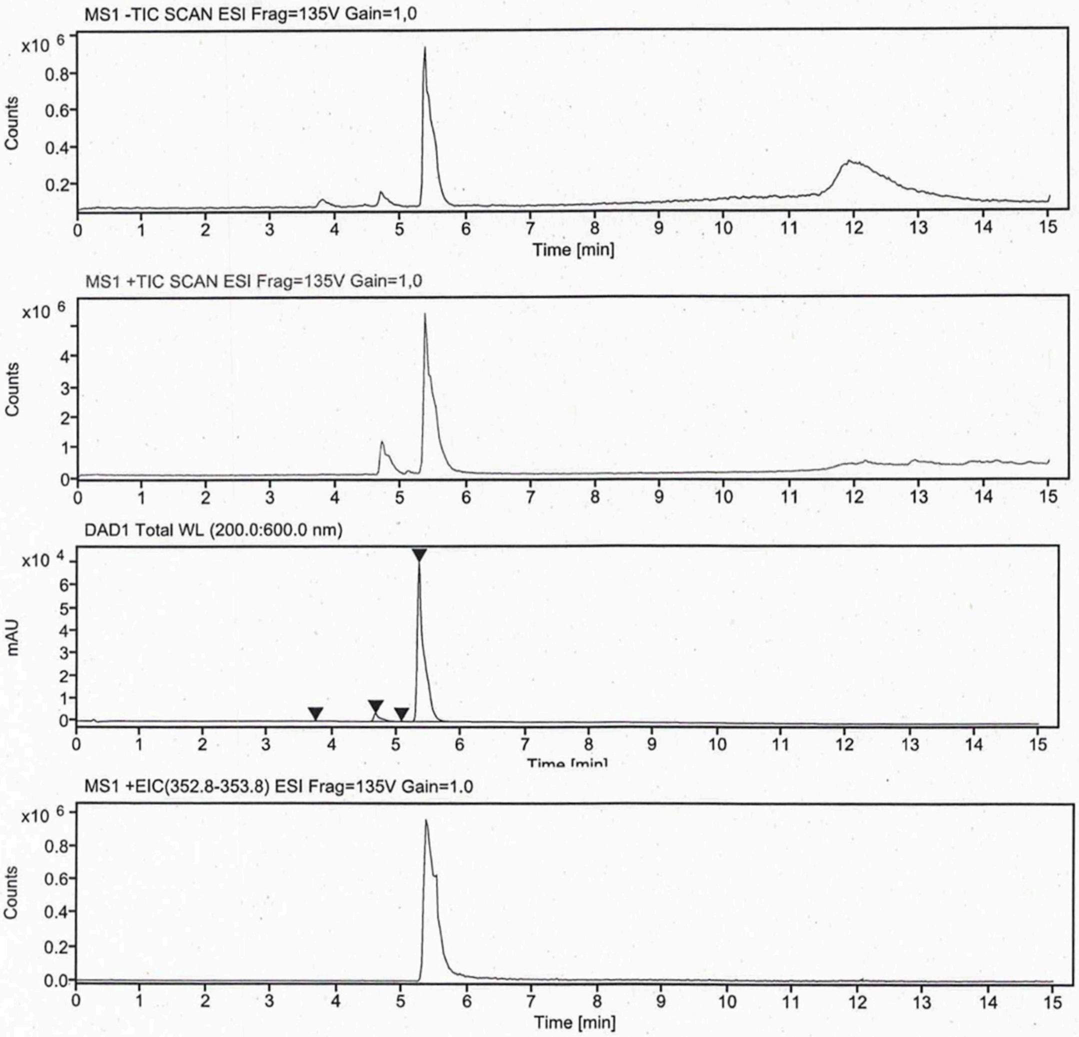

Signal: DAD1 Total WL (200.0:600.0 nm)

RT [min]	Peak MS Base Peak m/z	Area	Area%	Max Peak%	Height
3.738		1025.1785	0.1960	0.206	123.720
4.664	729.300	23398.8089	4.4746	4.697	3395.120
5.071		352.4711	0.0674	0.071	83.495
5.339	705.200	498151.6358	95.2620	100.000	69821.129
	Sum	522928.0943			

Signal: MS1 +TIC SCAN ESI Frag=135V Gain=1,0

RT [min]	Peak MS Base Peak m/z	Area	Area%	Max Peak%	Height
4.706	729.300	9367794.7218	14.9699	17.605	1071947.823
5.370	705.200	53209578.1978	85.0301	100.000	5418473.746
	Sum	62577372.9196			

Signal: MS1 -TIC SCAN ESI Frag=135V Gain=1,0

RT [min]	Peak MS Base Peak m/z	Area	Area%	Max Peak%	Height
5.373	351.200	8434114.0619	100.0000	100.000	868096.908
	Sum	8434114.0619			

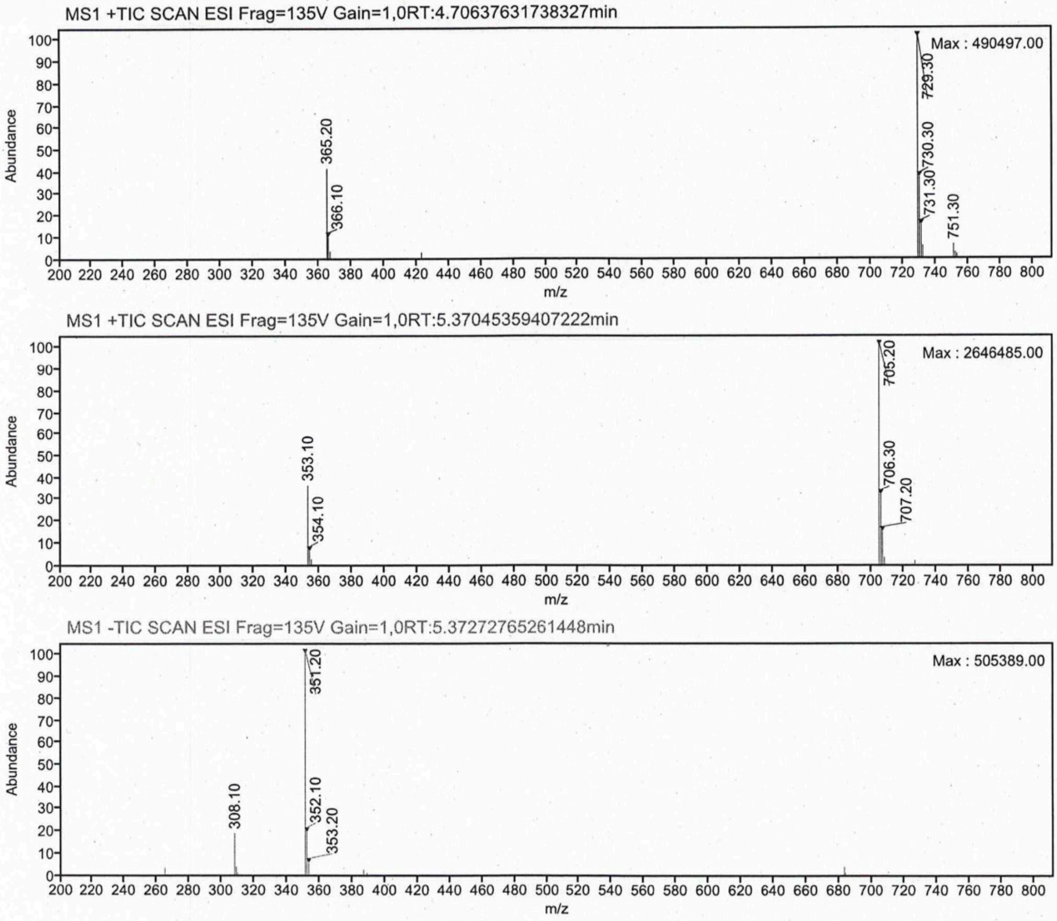

Figure S5. LC-MS spectrum of compound **6a***
*The purity of the compound **6a** is 95.26% (retention time: 5.34 min belongs to the desired compound **6a**; also see NMR spectra, **Figure S1**).

Inj. volume: 6.000 **Location:** P1-F1

Acq. method: 00-new_wasser-acn_standard_100-1000_BD-20min.amx

Processing method: MS_standard_plot.pmx

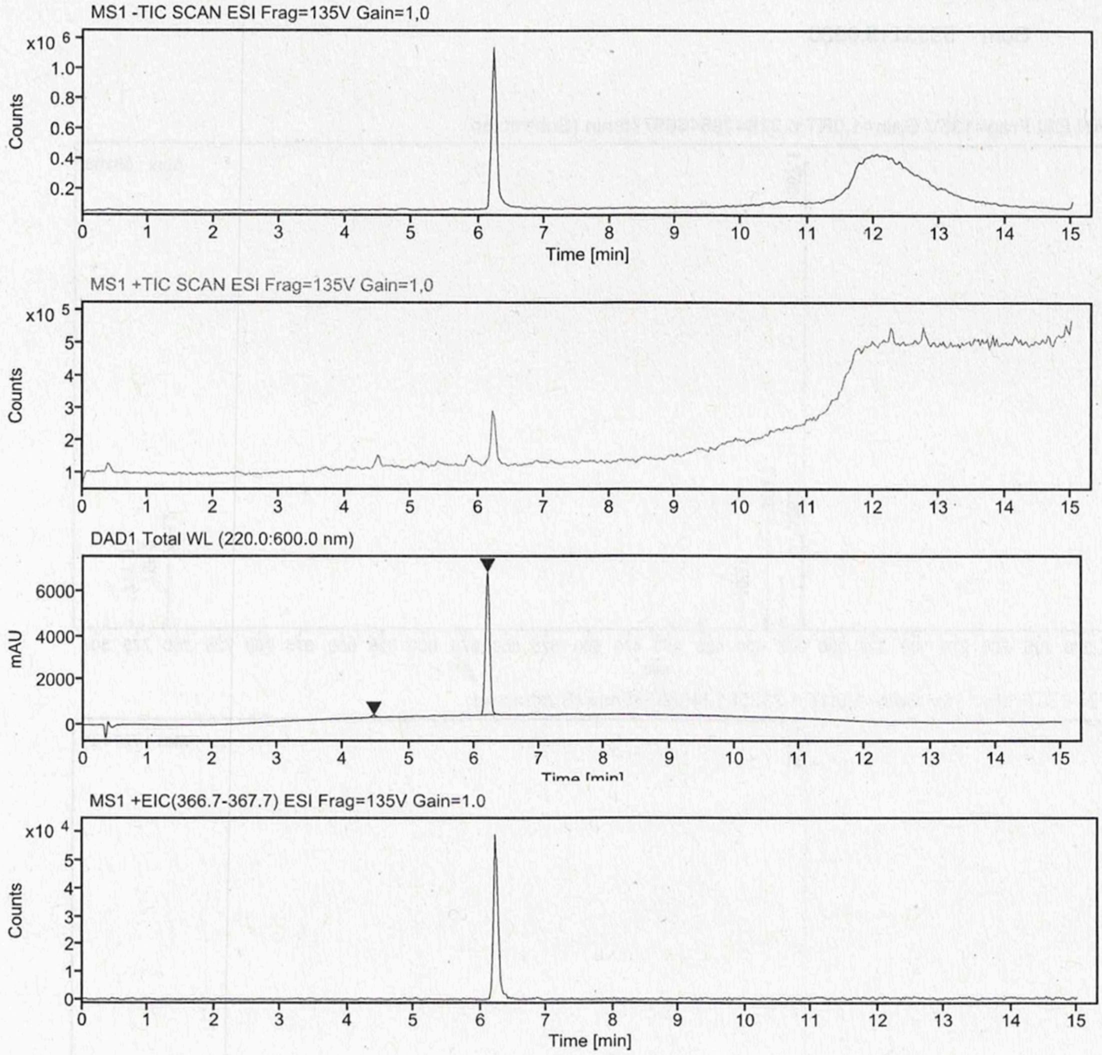

Signal: DAD1 Total WL (220.0:600.0 nm)

RT [min]	Peak MS Base Peak m/z	Area	Area%	Max Peak%	Height
4.459		405.3203	1.3315	1.349	91.380
6.200	367.100	30035.0161	98.6685	100.000	6432.036
	Sum	30440.3363			

Signal: MS1 +TIC SCAN ESI Frag=135V Gain=1,0

RT [min]	Peak MS Base Peak m/z	Area	Area%	Max Peak%	Height
6.228	367.100	1113401.2832	100.0000	100.000	184441.225
	Sum	1113401.2832			

Signal: MS1 -TIC SCAN ESI Frag=135V Gain=1,0

RT [min]	Peak MS Base Peak m/z	Area	Area%	Max Peak%	Height
6.234	365.100	5933119.0930	100.0000	100.000	1063015.152
	Sum	5933119.0930			

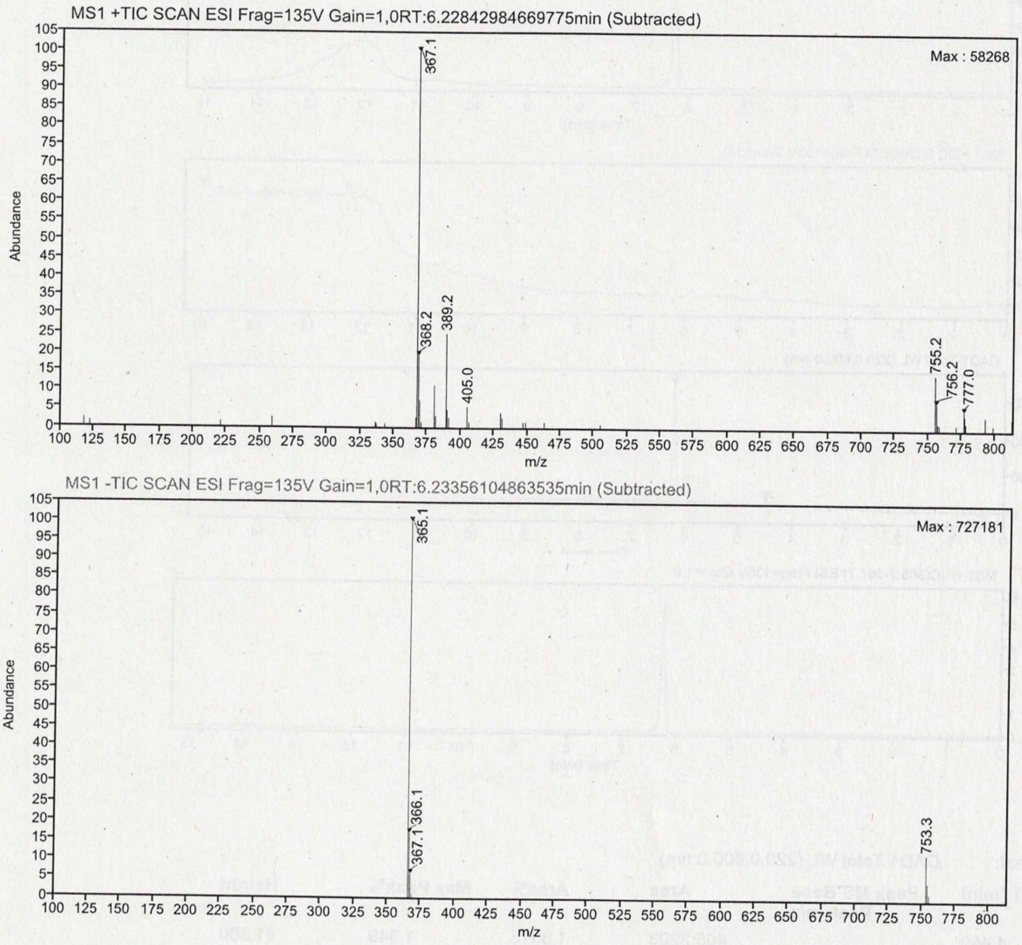

Figure S6. LC-MS spectrum of compound **6b***
*The purity of the compound **6b** is 98.67% (retention time: 6.20 min belongs to the desired compound **6b**; also see NMR spectra, **Figure S2**).

Inj. volume: 3.000 **Location:** P1-D2

Acq. method: 00-new_wasser-acn_standard_100-1000_BD-20min.amx

Processing method: MS_standard_plot.pmx

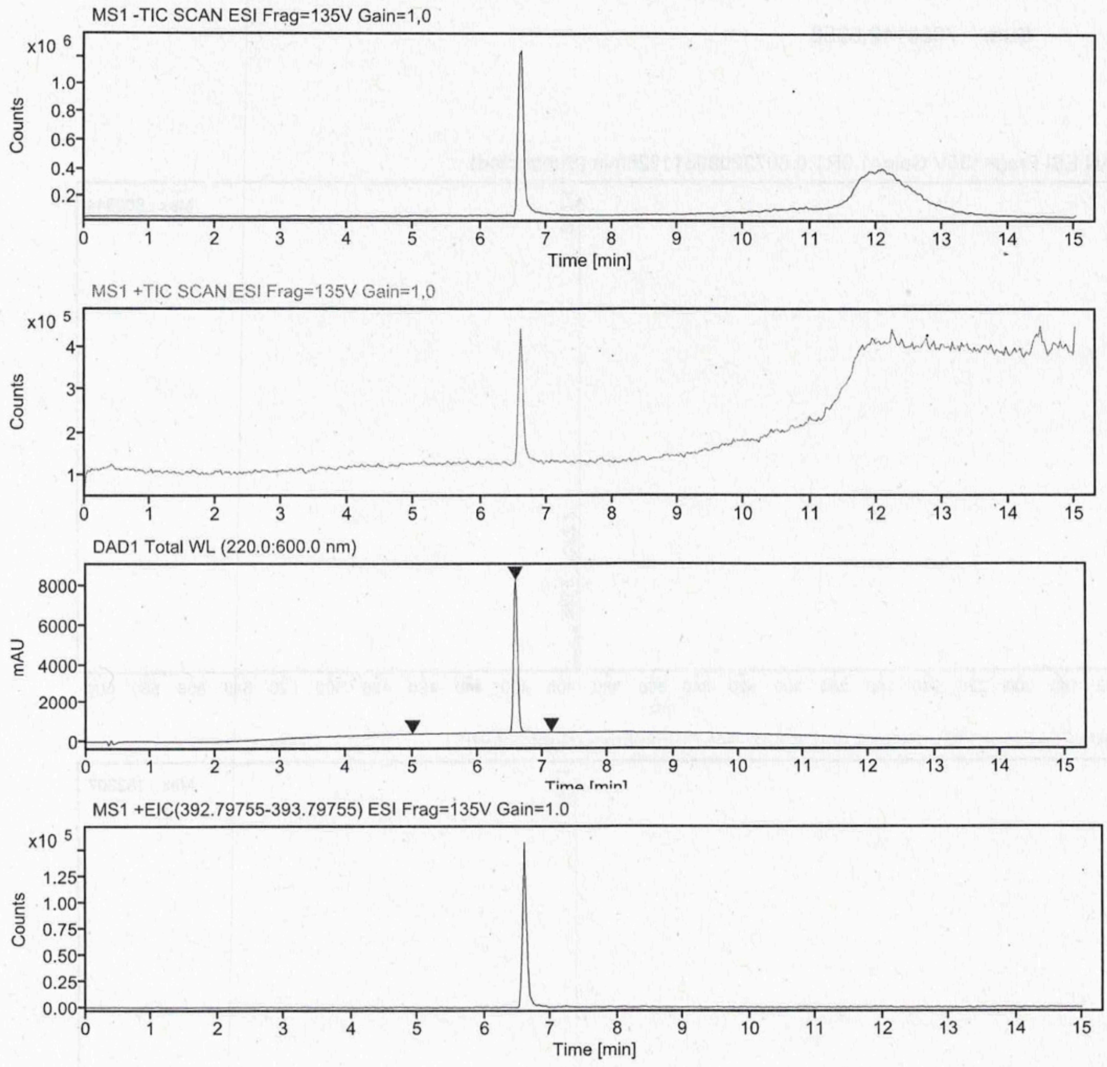

Signal: DAD1 Total WL (220.0:600.0 nm)

RT [min]	Peak MS Base Peak m/z	Area	Area%	Max Peak%	Height
5.004		23.2721	0.0660	0.066	5.976
6.577	391.100	35205.6266	99.8769	100.000	7842.946
7.124		20.1052	0.0570	0.057	4.478
	Sum	35249.0039			

Signal: MS1 +TIC SCAN ESI Frag=135V Gain=1,0

RT [min]	Peak MS Base Peak m/z	Area	Area%	Max Peak%	Height
6.608	393.100	1638656.7712	100.0000	100.000	316139.622
	Sum	1638656.7712			

Signal: MS1 -TIC SCAN ESI Frag=135V Gain=1,0

RT [min]	Peak MS Base Peak m/z	Area	Area%	Max Peak%	Height
6.607	391.100	7056142.6958	100.0000	100.000	1211529.400
	Sum	7056142.6958			

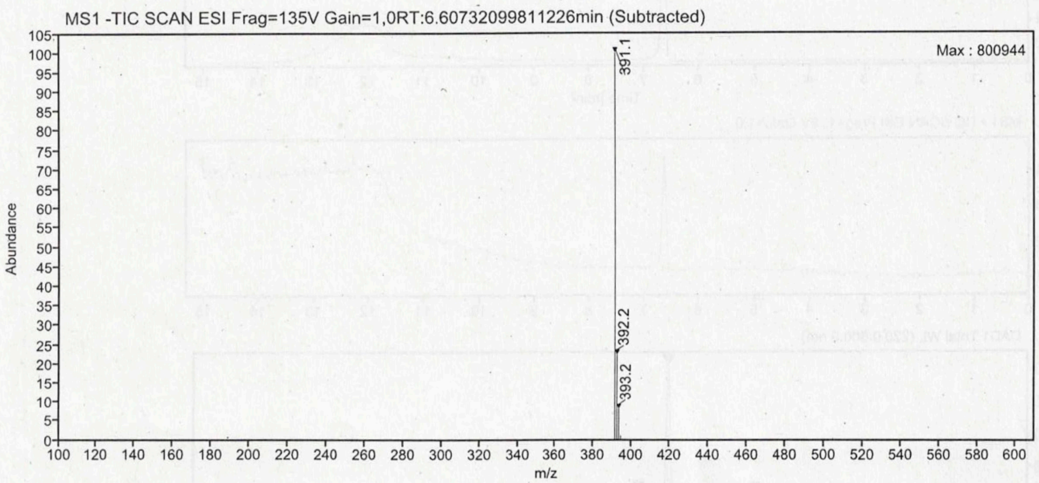

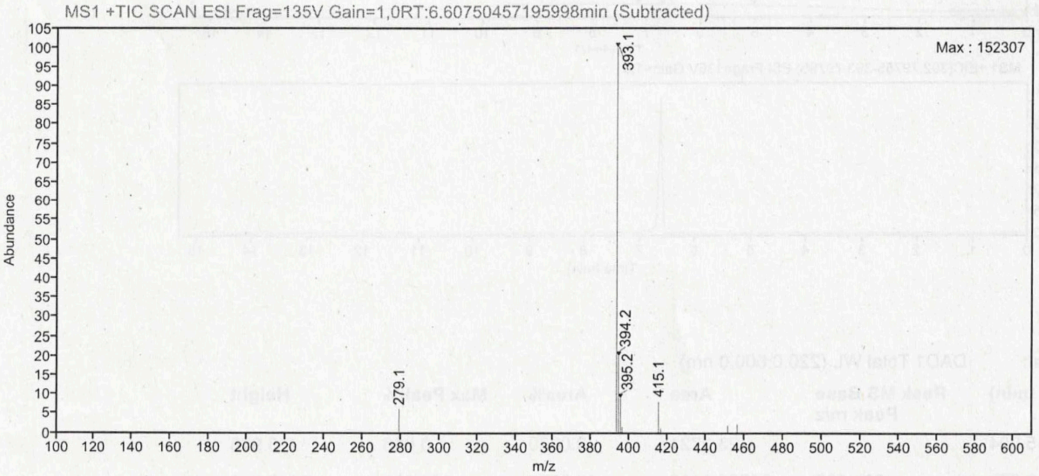

Figure S7. LC-MS spectrum of compound **6c***
*The purity of the compound **6c** is 99.88% (retention time: 6.58 min belongs to the desired compound **6c**; also see NMR spectra, **Figure S3**).

**Inj. volume:**	5.000	**Location:**	P1-E8
**Acq. method:**	00-new_wasser-acn_standard_100-1500_BD-20min.amx		
**Processing method:**	MS_standard_plot.pmx		

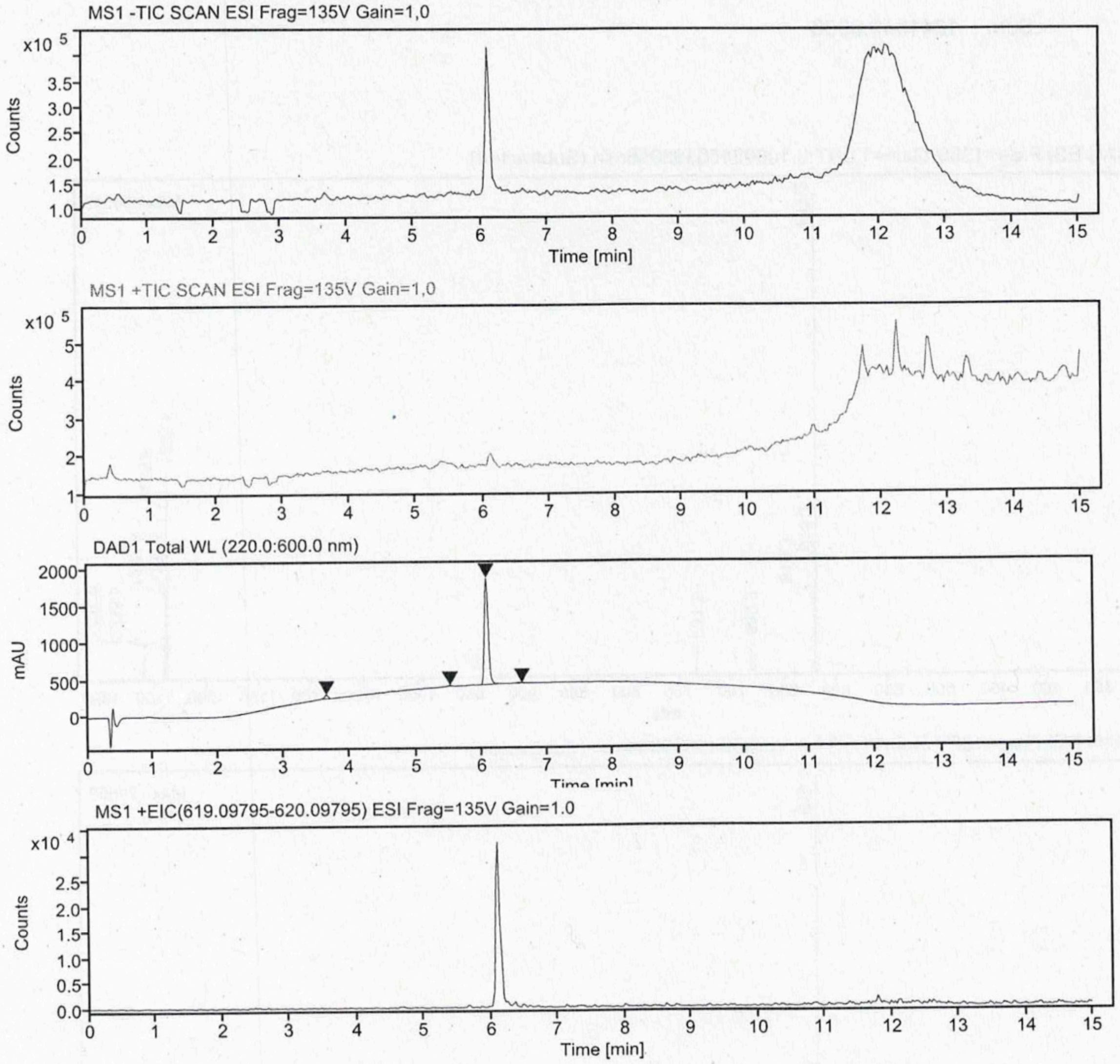

Signal: DAD1 Total WL (220.0:600.0 nm)

RT [min]	Peak MS Base Peak m/z	Area	Area%	Max Peak%	Height
3.647		234.1987	3.6323	3.810	58.064
5.518		17.2680	0.2678	0.281	6.499
6.075	617.300	6146.1972	95.3255	100.000	1448.974
6.608		49.9230	0.7743	0.812	6.926
	Sum	6447.5869			

Signal: MS1 +TIC SCAN ESI Frag=135V Gain=1,0

RT [min]	Peak MS Base Peak m/z	Area	Area%	Max Peak%	Height
6.112	619.400	170523.1399	100.0000	100.000	33217.708
	Sum	170523.1399			

Signal: MS1 -TIC SCAN ESI Frag=135V Gain=1,0

RT [min]	Peak MS Base Peak m/z	Area	Area%	Max Peak%	Height
6.106	617.300	1341846.8638	100.0000	100.000	294069.246
	Sum	1341846.8638			

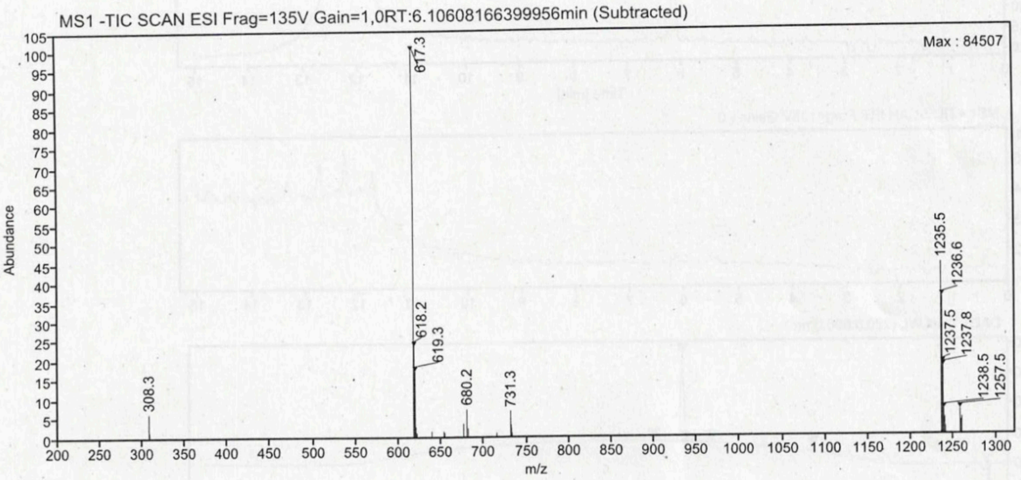

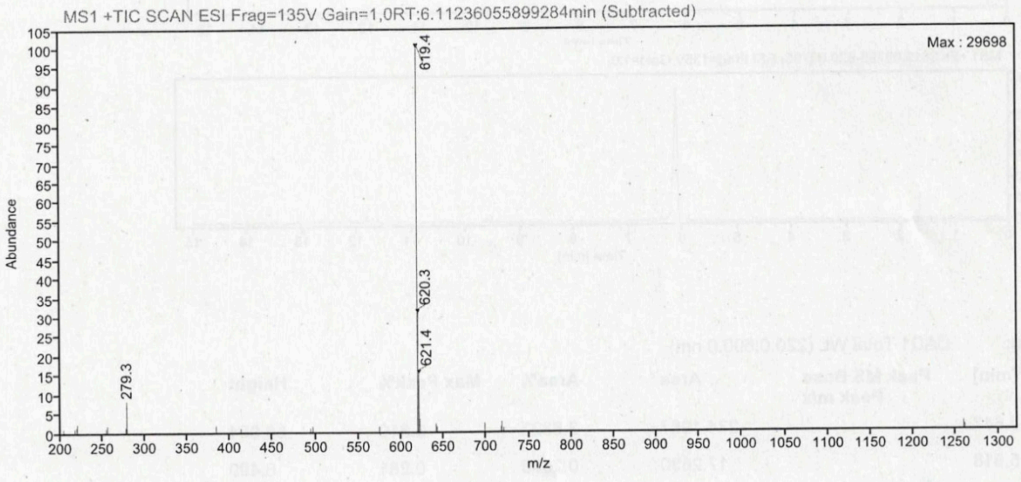

Figure S8. LC-MS spectrum of compound **12***
*The purity of the compound **12** is 95.33% (retention time: 6.08 min belongs to the desired compound **12**; also see NMR spectra, **Figure S4**).
